# Control of T_reg_ cell homeostasis and immune equilibrium by Lkb1 in dendritic cells

**DOI:** 10.1038/s41467-018-07545-8

**Published:** 2018-12-13

**Authors:** Song Chen, Lijun Fang, Wei Guo, Yushan Zhou, Gang Yu, Wenwen Li, Kui Dong, Jingru Liu, Yuechen Luo, Bing Wang, Zhonglong Li, Chunxiao Zhao, Zhina Sun, Yue Shen, Qibing Leng, Dongming Zhou, Zhongchao Han, Huifang Huang, He Ren, Guogang Xu, Xiaoming Feng

**Affiliations:** 1 State Key Laboratory of Experimental Hematology, Institute of Hematology and Hospital of Blood Disease, Chinese Academy of Medical Sciences & Peking Union Medical College, Tianjin, 300020 China; 2 Department of Respiration, The Second Affiliated Hospital of Nanchang University, Nanchang, 330006 China; 3 Central Laboratory, The Union Hospital of Fujian Medical University, Fuzhou, 350001 China; 4 Institute Pasteur of Shanghai, Shanghai Institute for Biological Sciences, Chinese Academy of Sciences, Shanghai, 200031 China; 5 Beijing Health & Biotech Group Corp. Ltd., No. 1 Kangding Road BDA, Beijing, 100176 China; 6 Department of Pancreatic Cancer, Key Laboratory of Cancer Prevention and Therapy, Tianjin Medical University Cancer Institute and Hospital, Tianjin, 300060 China; 7 Nanlou Respiratory Department, Chinese PLA General Hospital, Beijing, 100039 China; 8 Key Laboratory of Immune Microenvironment and Disease of the Ministry of Education, Tianjin Medical University, Tianjin, 300070 China

## Abstract

To balance immunity and tolerance, the endogenous pool of Foxp3^+^ regulatory T (T_reg_) cells is tightly controlled, but the underlying mechanisms of this control remain poorly understood. Here we show that the number of T_reg_ cells is negatively regulated by the kinase Lkb1 in dendritic cells (DCs). Conditional knockout of the *Lkb1* gene in DCs leads to excessive T_reg_ cell expansion in multiple organs and dampens antigen-specific T cell immunity. Lkb1-deficient DCs are capable of enhancing, compared with wild-type DCs, T_reg_ cell proliferation via cell-cell contact involving the IKK/IKBα-independent activation of the NF-κB/OX40L pathway. Intriguingly, treating wild-type mice with lipopolysaccharide selectively depletes Lkb1 protein in DCs, resulting in T_reg_ cell expansion and suppressed inflammatory injury upon subsequent challenge. Loss of Lkb1 does not obviously upregulate proinflammatory molecules expression on DCs. We thus identify Lkb1 as a regulatory switch in DCs for controlling T_reg_ cell homeostasis, immune response and tolerance.

## Abstract (web summary)

Regulatory T (Treg) cells are important for maintaining immune homeostasis by suppressing immune cell activation, but how the Treg cell pool is maintained is still unclear. Here the authors show that a kinase, Lkb1, operates in dendritic cells (DC) to inhibit Treg cell expansion and immunosuppression via mechanisms involving NF-kB/OX40L signalling.

## Introduction

Foxp3^+^ T_reg_ cells, either derived from the thymus (natural T_reg_ cells, nT_reg_ cells) or induced from conventional T (T_con_) cells in the periphery (induced T_reg_ cells, iT_reg_ cells), play pivotal roles in suppressing immune responses^1,2^. Under steady-state conditions, the number of T_reg_ cells in each organ is maintained at a constant threshold level to ensure self-tolerance while allowing the efficient initiation of defensive responses^2^. During a variety of inflammatory processes, T_reg_ cells usually increase in number through proliferation or de novo generation, and this increase might serve as critical negative feedback to restrain inflammatory injuries^1,3,4^. However, despite the earlier recognition of IL-2 produced by effector T cells as a basic factor in the maintenance of the T_reg_ cell pool^1,2^, our knowledge of how the number of T_reg_ cells is specifically regulated in a wide range of tissues and under various immune conditions is still very limited.

Originally known as sentinels of the immune system required for initiating defensive responses, DCs critically maintain immune homeostasis not only through suppressing the activation or inducing the unresponsiveness/apoptosis of self-reactive T cells but also by promoting the generation, maintenance and/or expansion of T_reg_ cells^5–7^. It is widely accepted that DCs can induce the de novo generation of iT_reg_ cells via mechanisms dependent on antigen presentation to induce a distinct T cell receptor (TCR) signal^8^, costimulation via B7-CTLA4^9^ or BTLA-HVEM^10^ ligation, and TGF-β^11^ and retinoic acid^12–14^ production. Nevertheless, how DCs maintain or expand the existing pool of T_reg_ cells to balance immunity and tolerance in vivo is unclear.

Liver kinase B1 (Lkb1) is a serine-threonine kinase that was first identified as a tumour suppressor whose mutation is responsible for Peutz Jeghers syndrome^15,16^. Previous studies have highlighted a prominent role played by Lkb1 in the immune system; Lkb1 has been identified as a critical regulator of T cell development, activation, and metabolism^17^. Our recent work indicates that Lkb1 epigenetically stabilizes Foxp3 expression and promotes suppressive functions in T_reg_ cells^18^. Lkb1 also inhibits the activation and inflammatory functions of innate macrophages^19^. However, the specific role played by Lkb1 in DCs, which are central to immune regulation, has not yet been studied.

In this study, using mice with the *Lkb1* gene conditionally deleted in DCs, we find that the expression of Lkb1 is a feedforward factor in DCs rather than other immune cell types, required for restraining the steady-state T_reg_ cell numbers in multiple organs, thereby allowing the efficient initiation of antigen-specific immune responses. We further evaluate whether ectopic Lkb1 downregulation in DCs contributes to increases in the T_reg_ cell population during inflammatory processes. Indeed, we find that after challenging wild-type (WT) mice with *Escherichia coli* (*E. coli*)-derived lipopolysaccharide (LPS), Lkb1 protein is selectively depleted in DCs in a negative-feedback manner, resulting in the expansion of T_reg_ cells that is necessary for protecting the host from inflammatory injury responses induced by lethal re-challenge doses. Mechanistically, the loss of Lkb1 activates a regulatory transcriptional programme in DCs, including the upregulation of *Ox40l* gene expression, to stimulate T_reg_ cell proliferation. Thus, we provide data indicating that the homeostasis of T_reg_ cells and the strength of immunosuppression are dynamically controlled by an Lkb1 regulatory switch in DCs, in a feedback manner, to ensure immune equivalence.

## Results

### Lkb1 deletion in DCs leads to T_reg_ pool enlargement

To investigate the role of Lkb1 in DCs, we generated a line of mice with *Lkb1* conditionally deleted in DCs by crossing *Cd11c*^Cre^ mice with *Lkb1*^f/f^ mice. We analysed the phenotype of DCs in the spleen and lymph nodes (LNs). No significant differences in the population size or cell surface expression of activation markers, including major histocompatibility complex (MHC) class II molecules and co-stimulatory molecules CD80 and CD86, were observed between DCs from *Lkb1*^f/f^ and *Cd11c*^Cre^*Lkb1*^f/f^ mice (Supplementary Fig. 1a-c), indicating that Lkb1 has little effect on DC maintenance and activation.

Since the major functions of DCs are to preserve steady-state T cell homeostasis and induce foreign antigen-specific T cell responses^20^, we sought to determine the phenotypical and functional alterations in T cells from *Lkb1*^f/f^ and *Cd11c*^Cre^*Lkb1*^f/f^ mice. *Cd11c*^Cre^*Lkb1*^f/f^ mice had higher percentages of CD4^+^Foxp3^-^ and CD8^+^Foxp3^-^ T cells that exhibited a CD44^hi^CD62L^lo^ effector/memory phenotype (Supplementary Fig. 2a). However, despite a slight elevation in IL-2 and IL-17 production, the IL-4 and IFN-γ levels were not significantly increased in CD4^+^Foxp3^-^ or CD8^+^Foxp3^-^ T cells from *Cd11c*^Cre^*Lkb1*^f/f^ mice (Supplementary Fig. 2b), indicating that excessive effector T cell differentiation was not present in *Cd11c*^Cre^*Lkb1*^f/f^ mice. Indeed, the *Cd11c*^Cre^*Lkb1*^f/f^ mice exhibited a normal body weight (Supplementary Fig. 2c) and did not display any signs of autoimmunity (Supplementary Fig. 2d). These results indicate that *Cd11c*^Cre^*Lkb1*^f/f^ mice are devoid of autoimmune diseases, even in the presence of a slightly activated T cell phenotype. Surprisingly, there were no significant differences in the proliferation of CFSE-labelled CD4^+^ and CD8^+^ T cells sorted from OT-II and OT-I transgenic mice after being co-cultured with *Lkb1*^f/f^ and *Cd11c*^Cre^*Lkb1*^f/f^ DCs loaded with the respective antigenic peptide in vitro (Supplementary Fig. 2e), suggesting that Lkb1 does not affect the DC function of priming antigen-specific T cell responses.

**Fig. 1:**
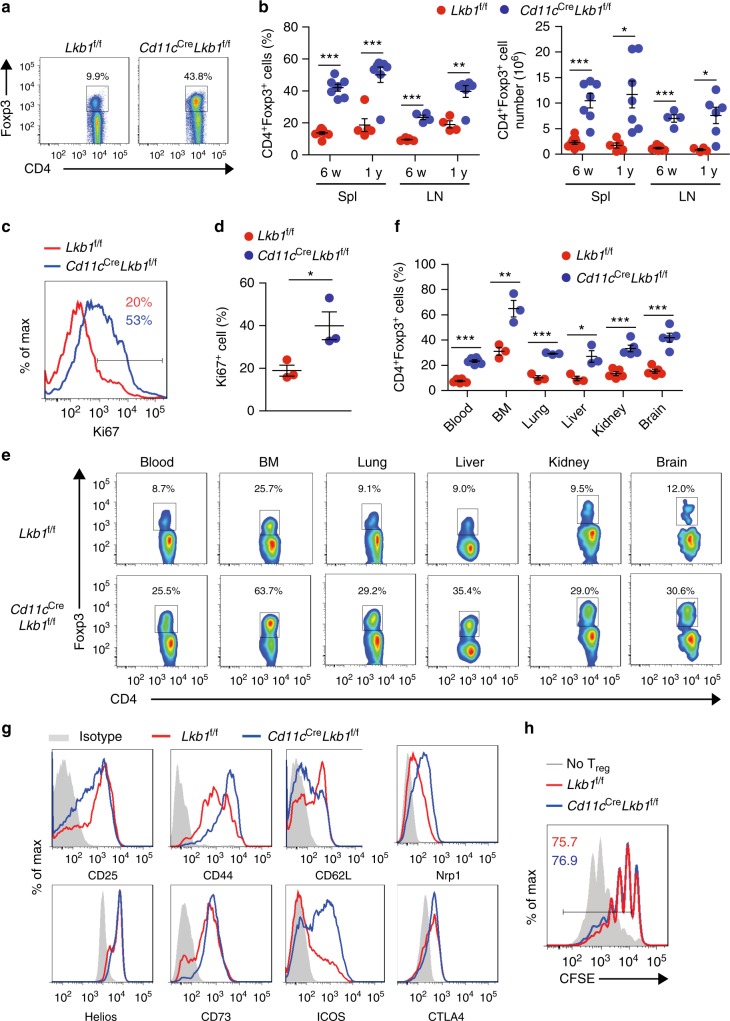
DC-specific deletion of Lkb1 leads to specific enlargement of T_reg_ cell pool. **a** Flow cytometric analysis of T_reg_ cell frequencies among splenic CD4^+^ T cells from *Lkb1*^f/f^ and *Cd11c*^Cre^*Lkb1*^f/f^ mice. **b** Quantification of the percentage and absolute number of T_reg_ cells in the spleen and lymph nodes (LNs) from *Lkb1*^f/f^ (red dots) and *Cd11c*^Cre^*Lkb1*^f/f^ (blue dots) mice of different ages. **c**, **d** Flow cytometric analysis (**c**) and quantification (**d**) of Ki67 expression in splenic T_reg_ cells from *Lkb1*^f/f^ and *Cd11c*^Cre^*Lkb1*^f/f^ mice. **e** Flow cytometric analysis of T_reg_ cell frequencies among CD4^+^ T cells in the blood, bone marrow (BM), lungs, liver, kidneys and brain of *Lkb1*^f/f^ and *Cd11c*^Cre^*Lkb1*^f/f^ mice. **f** Quantification of the percentages of T_reg_ cells among CD4^+^ T cells in the blood, BM, lungs, liver, kidneys and brain of *Lkb1*^f/f^ (red dots) and *Cd11c*^Cre^*Lkb1*^f/f^ (blue dots) mice. **g** Flow cytometric analysis of CD25, CD44, CD62L, Helios, CD73, ICOS, Nrp1 and CTLA4 expression on splenic T_reg_ cells from *Lkb1*^f/f^ (red line) and *Cd11c*^Cre^*Lkb1*^f/f^ (blue line) mice. **h** Suppression assay in which CFSE-labelled naïve T (T_n_) cells were co-cultured with T_reg_ cells from *Lkb1*^f/f^ and *Cd11c*^Cre^*Lkb1*^f/f^ mice at a 4:1 ratio. Each symbol (**b**, **f**) indicates an individual mouse; the results are presented as the mean ± S.E.M., *P < 0.05, **P < 0.01, ***P < 0.001, by Student's *t*-test (**b**, **d**, **f**). Data are pooled from **b**, **d**, **f** or are representative of **a**, **c**, **e**, **g**, **h** three independent experiments with similar results

Strikingly, the percentage and absolute number of Foxp3^+^ T_reg_ cells were greatly increased in the spleen and LNs from *Cd11c*^Cre^*Lkb1*^f/f^ mice compared with those from *Lkb1*^f/f^ mice, as was the expression of Ki67, an indicator of cell proliferation (Fig. 1a–d). However, no significant difference in the apoptosis of T_reg_ cells from *Lkb1*^f/f^ mice and *Cd11c*^Cre^*Lkb1*^f/f^ was observed (Supplementary Fig. 3a, b). There were also significantly higher frequencies of T_reg_ cells in peripheral non-lymphoid organs, including the blood, bone marrow (BM), lungs, liver, kidneys and brain, from *Cd11c*^Cre^*Lkb1*^f/f^ mice than in those from *Lkb1*^f/f^ mice (Fig. 1e, f). However, the T_reg_ cell frequency was not significantly elevated in the thymus in *Cd11c*^Cre^*Lkb1*^f/f^ mice (Supplementary Fig. 3c, d), suggesting that the enlarged T_reg_ cell compartment in the periphery was not caused by developmental dysregulation in the thymus. T_reg_ cells from *Cd11c*^Cre^*Lkb1*^f/f^ mice displayed a CD44^hi^CD62L^lo^ activated phenotype and expressed higher levels of activation markers CD73 and ICOS (Fig. 1g). In addition, T_reg_ cells from *Cd11c*^Cre^*Lkb1*^f/f^ mice expressed high levels of Nrp1 and Helios^21–23^, which are characteristic markers of nT_reg_ cells (Fig. 1g), suggesting that these expanded T_reg_ cells were mainly derived from the thymus rather than T_con_ cells in the periphery. Thus, the Lkb1 deficiency in DCs caused strong T_reg_ cell expansion in the periphery. However, Lkb1 seemed not to directly affect the suppressor function of T_reg_ cells, since there was no difference in the suppressive capacity of T_reg_ cells from *Lkb1*^f/f^ and *Cd11c*^Cre^*Lkb1*^f/f^ mice (Fig. 1h).

*Cd11c*^Cre^*Lkb1*^f/f^ mice lack Lkb1 in all cells expressing CD11c, which include DCs and macrophages. To determine whether Lkb1-deficient macrophages contribute to the phenotype of the mice, we examined T_reg_ and T_con_ cells in the spleen and LNs of *Lkb1*^f/f^ and *LysM*^Cre^*Lkb1*^f/f^ mice, which carry the specific deletion of *Lkb1* in myeloid-derived cells, including macrophages. We found that the Lkb1 deficiency in macrophages did not affect the percentages of T_reg_ cells or the activation of CD4^+^ Foxp3^−^ and CD8^+^ Foxp3^−^ T cells (Supplementary Fig. 4a-d). These results further confirm that expansion of the T_reg_ compartment was mainly caused by the deletion of *Lkb1* in DCs.

### Impaired immune responses due to increased T_reg_ cell number

**Fig. 2:**
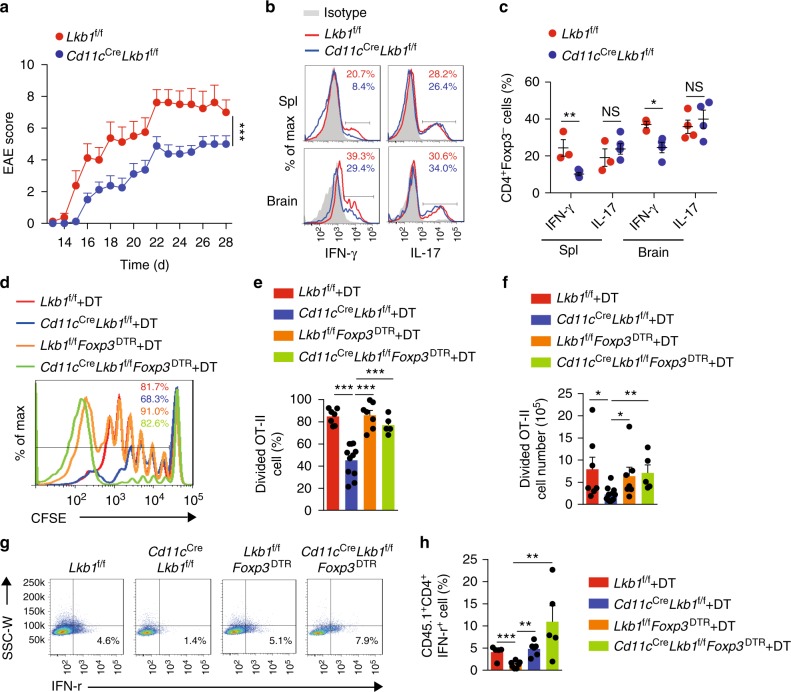
Impaired adaptive immune responses due to increased T_reg_ cell number. **a** Disease-related clinical scores of *Lkb1*^f/f^ and *Cd11c*^Cre^*Lkb1*^f/f^ mice subjected to MOG35–55-induced experimental autoimmune encephalomyelitis (EAE). **b**, **c** Flow cytometric analysis (**b**) and quantification (**c**) of IFN-γ- and IL-17-producing CD4^+^Foxp3^−^ T cells in the spleen and brain of *Lkb1*^f/f^ and *Cd11c*^Cre^*Lkb1*^f/f^ mice with EAE. **d**–**f** Flow cytometric analysis (**d**), percentage (**e**) and absolute number (**f**) of CFSE-labelled CD4^+^ T cells (2×10^6^) from OT-II mice transferred into *Lkb1*^f/f^, *Cd11c*^Cre^*Lkb1*^f/f^, *Lkb1*^f/f^*Foxp3*^DTR^ and *Cd11c*^Cre^*Lkb1*^f/f^*Foxp3*^DTR^ mice treated with diphtheria toxin (DT) (50μgkg^−1^, 2 days in advance). **g**, **h** Flow cytometric analysis (**g**) and quantification (**h**) of IFN-γ-producing OT-II T cells in *Lkb1*^f/f^, *Cd11c*^Cre^*Lkb1*^f/f^, *Lkb1*^f/f^*Foxp3*^DTR^ and *Cd11c*^Cre^*Lkb1*^f/f^*Foxp3*^DTR^ mice stimulated with phorbol myristate acetate (PMA) and ionomycin for 4 h. The results are presented as the mean ± S.E.M., *P < 0.05, **P < 0.01, by two-way ANOVA (**a**) or Student's *t*-test (**c**, **e**, **f**, **h**). Data are pooled from **c**, **e**, **f, h** or are representative of **a**, **b**, **d**, **g** three independent experiments with similar results

T_reg_ cells play a predominant role in suppressing immune responses in vivo. We next determined the impact of the increased T_reg_ cell number on antigen-specific T cell responses using an experimental autoimmune encephalomyelitis (EAE) mouse model. We observed lower clinical scores, reflecting less severe EAE, in *Cd11c*^Cre^*Lkb1*^f/f^ mice than in WT littermates (Fig. 2a). It is well known that EAE is mediated by encephalitogenic Th1 and Th17 cells, which produce pro-inflammatory cytokines IFN-γ and IL-17, respectively^24^. Thus, we analysed the populations of CD4^+^IFN-γ^+^ T (Th1) and CD4^+^IL-17^+^ T (Th17) cells in the spleen and brain. In *Cd11c*^Cre^*Lkb1*^f/f^ mice, the Th1 cell populations, but not the Th17 populations, were significantly decreased in the spleen and brain (Fig. 2b, c). The levels of IL-17-producing T cells were slightly increased in *Cd11c*^Cre^*Lkb1*^f/f^ mice under steady-state conditions (Supplementary Fig. 2b), which indicates that Lkb1-deficient DCs might intrinsically have an increased capacity for stimulating Th17 differentiation. There was no difference in the extent of Th17 cell development between the *Lkb1*^f/f^ and *Cd11c*^Cre^*Lkb1*^f/f^ mice in the EAE model, suggesting that enhanced Th17 differentiation might in some manner counteract the enhanced suppressor function of the expanded T_reg_ population. However, significantly reduced Th1 cell development and EAE disease severity were observed in *Cd11c*^Cre^*Lkb1*^f/f^ mice, indicating that the expanded T_reg_ cell population dominated and resulted in overall alleviation of the immune response. As Lkb1 deficiency barely affected the antigen-specific T cell priming function of DCs (Supplementary Fig. 2e), we speculated that the impaired Th1 response was most likely due to enhanced immune suppression mediated by the enlarged T_reg_ cell compartment in *Cd11c*^Cre^*Lkb1*^f/f^ mice. To further confirm the impact of increased T_reg_ cell populations on antigen-specific T cell responses, we bred *Foxp3*^DTR^ mice to generate *Lkb1*^f/f^*Foxp3*^DTR^ and *Cd11c*^Cre^*Lkb1*^f/f^*Foxp3*^DTR^ mice, in which Foxp3^+^ T_reg_ cells could be specifically depleted with diphtheria toxin (DT) treatment (short-term DT treatment only resulted in a transient reduction in the T_reg_ cell populations and did not lead to overt disease phenotypes or death in mice)^25^, and we conducted an *in vivo* assay of OVA antigen-induced T cell priming. Two days after DT treatment (Supplementary Fig. 5a), we adoptively transferred CFSE-labelled CD4^+^ T cells from OT-II transgenic mice into DT-treated *Lkb1*^f/f^, *Cd11c*^Cre^*Lkb1*^f/f^, *Lkb1*^f/f^*Foxp3*^DTR^ and *Cd11c*^Cre^*Lkb1*^f/f^*Foxp3*^DTR^ mice via a tail vein injection, and we then challenged these mice with indicated doses of OVA to induce an antigen-specific T cell response. Lower percentages and numbers of proliferating OT-II T cells were detected in *Cd11c*^Cre^*Lkb1*^f/f^ mice than in *Lkb1*^f/f^ mice, while OT-II T cells in *Cd11c*^Cre^*Lkb1*^f/f^*Foxp3*^DTR^ mice treated with DT exhibited stronger proliferation and higher absolute numbers than OT-II T cells in *Cd11c*^Cre^*Lkb1*^f/f^ mice (Fig. 2d–f). In addition, the production of IFN-γ in OT-II cells was negatively correlated with the proportion of T_reg_ cells in vivo (Fig. 2g, h). These data suggest that the impaired T cell response in the *Cd11c*^Cre^*Lkb1*^f/f^ mice was due to the expanded T_reg_ cell compartment.

### Lkb1-deficient DCs promote T_reg_ cell proliferation

**Fig. 3:**
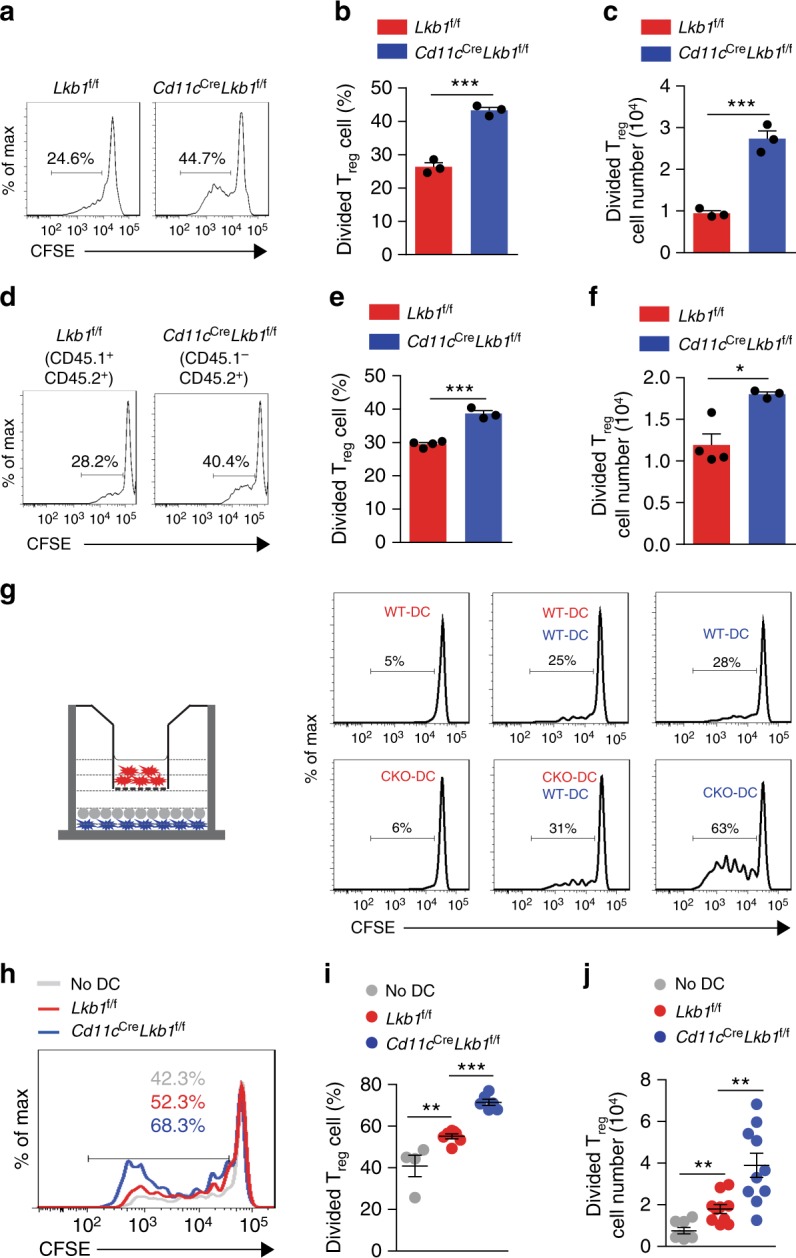
Lkb1-deficient DCs directly stimulate T_reg_ cell proliferation. **a**–**c** Flow cytometric analysis (**a**), percentage (**b**) and number (**c**) of CFSE-labelled T_reg_ cells (CD4^+^Foxp3^+^, 2×10^5^) sorted from B6 mice after co-culture with DCs (1×10^5^) sorted from *Lkb1*^f/f^ or *Cd11c*^Cre^*Lkb1*^f/f^ mice supplemented with IL-2 for 4 days. **d**–**f** Flow cytometric analysis (**d**), percentage (**e**) and number (**f**) of CFSE-labelled T_reg_ cells sorted from B6 mice after co-culture with WT or Lkb1-deficient DCs sorted from mixed BM chimaera mouse models. **g** Flow cytometric analysis of the proliferation of CFSE-labelled T_reg_ cells sorted from B6 mice in the lower Transwell chamber after 4 days of co-culture with DCs from *Lkb1*^f/f^ (wild-type, WT) and/or *Cd11c*^Cre^*Lkb1*^f/f^ (conditional knockout, CKO) mice in the upper and/or lower chamber of a Transwell plate, in diverse combinations, as indicated. In the schematic diagram, grey dots indicate T_reg_ cells, while red and blue dots indicate DCs in the upper and lower chamber, respectively. The coloured phrases in the histogram plots indicate the setup of DCs under each culture condition. **h**–**j** Flow cytometric analysis (**h**), percentage (**i**) and number (**j**) of CFSE-labelled T_reg_ cells (CD4^+^Foxp3^+^, 2×10^6^) sorted from B6 mice transferred together with or without DCs (1×10^6^) sorted from *Lkb1*^f/f^ or *Cd11c*^Cre^*Lkb1*^f/f^ mice into irradiated NSG mice via tail vein injection for 3 days. The results are presented as the mean ± S.E.M., *P < 0.05, by Student's *t*-test (**b**, **c**, **e**, **f**, **i**, **j**). Data are pooled from **b**, **c**, **i**, **j** or are representative of **a**, **d**, **g**, **h** three independent experiments

Although IL-2 plays a role in promoting T_reg_ cell proliferation, the increased production of IL-2 in CD4^+^Foxp3^−^ T cells in *Cd11c*^Cre^*Lkb1*^f/f^ mice was slight (Supplementary Fig. 2b) and could not explain the extremely high proportion of T_reg_ cells among CD4^+^ T cells. In addition, as the most prominent driving factor of effector T cell responses, the elevation of IL-2 alone could not account for the defective T cell response in the *Cd11c*^Cre^*Lkb1*^f/f^ mice. Therefore, we postulated that Lkb1-deficient DCs could directly promote T_reg_ cell proliferation. Since splenic DCs could facilitate ubiquitous or tissue-restricted self-antigen-mediated TCR signalling to induce the polyclonal expansion of T_reg_ cells^26,27^, we established a co-culture system to assess the direct impact of DCs on T_reg_ cells^18^. To determine the direct effect of Lkb1-deficient DCs on T_reg_ cell expansion, we sorted splenic DCs (CD11c^+^MHCII^+^) from *Lkb1*^f/f^ and *Cd11c*^Cre^*Lkb1*^f/f^ mice and co-cultured them with CFSE-labelled T_reg_ cells (CD4^+^CD25^+^ population containing more than 95% Foxp3^+^ cells) sorted from B6 (CD45.1^+^) mice in the presence of exogenous IL-2 (Supplementary Fig. 5b). The T_reg_ cells displayed markedly increased proliferation and higher numbers of CD4^+^Foxp3^+^ cells when co-cultured with Lkb1-deficient DCs than when co-cultured with WT DCs (Fig. 3a–c). We also confirmed this result with T_reg_ cells (CD4^+^YFP^+^) from *Foxp3*^YFP-Cre^ mice (Supplementary Fig. 5c-e). Next, we established a mixed BM chimaera mouse model to acquire WT and Lkb1-deficient DCs from the same environment. After co-culturing these cells with CFSE-labelled T_reg_ cells in the presence of exogenous IL-2 for 4 days, we observed the same result, Lkb1-deficient DCs could induce stronger T_reg_ cell expansion (Fig. 3d–f). These results indicate that Lkb1-deficient DCs could directly promote T_reg_ cell expansion independent of other environmental elements. We next used Transwell plates to explore whether this effect of DCs was dependent on direct cell–cell contact or soluble factors secreted from DCs; in this way, T_reg_ cells and DCs could be physically separated but share the same medium in close proximity. Indeed, we found that enhanced T_reg_ cell proliferation only occurred when direct contact was made with Lkb1-deficient DCs (Fig. 3g), suggesting that Lkb1-deficient DCs promoted T_reg_ cell proliferation in a contact-dependent manner. To determine whether Lkb1-deficient DCs could induce T_reg_ cell proliferation in vivo, we transferred DCs sorted from *Lkb1*^f/f^ or *Cd11c*^Cre^*Lkb1*^f/f^ mice together with T_reg_ cells into irradiated NSG mice. After 3 days, we detected that ~85% of the transferred cells maintained Foxp3 expression (Supplementary Fig. 5f), and a markedly greater absolute number of divided T_reg_ cells was observed after co-transfer with Lkb1-deficient DCs than co-transfer with WT DCs (Fig. 3h–j). These results indicate that Lkb1 depletion in DCs was a direct driver of T_reg_ cell proliferation.

### DC OX40L upregulation contributes to T_reg_ cell proliferation

AMPK is a well-known downstream Lkb1 target critical for regulating metabolism^28^. To determine whether AMPK is involved in Lkb1-deficient DC function, we generated *Cd11c*^Cre^*AMPKα1*^f/f^*AMPKα2*^f/f^ mice with the specific depletion of AMPK in DCs. However, there were no significant differences in the percentage of T_reg_ cells or the activation of T_con_ cells between *AMPKα1*^f/f^*AMPKα2*^f/f^ and *Cd11c*^Cre^*AMPKα1*^f/f^*AMPKα2*^f/f^ mice (Supplementary Fig. 6a-d), suggesting that Lkb1 function in DCs is independent of AMPK activation.

**Fig. 4:**
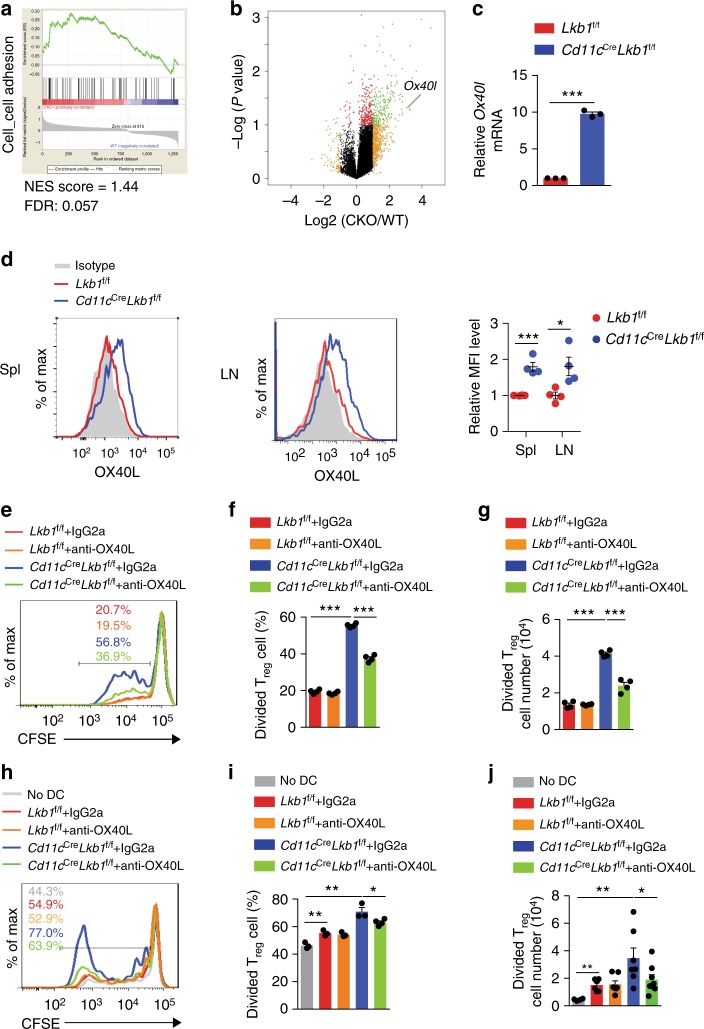
Lkb1-deficient DCs promote T_reg_ cell expansion through the OX40L-OX40 axis. **a** Gene set enrichment analysis (GSEA) of transcriptional profiles in WT and Lkb1-deficient DCs. The gene set of cell-cell adhesion was enriched in *Cd11c*^Cre^*Lkb1*^f/f^ DCs. FDR, false-discovery rate; NES, normalized enrichment score. **b** Volcano plot analysis of gene expression in splenic DCs from *Lkb1*^f/f^ and *Cd11c*^Cre^*Lkb1*^f/f^ mice. The points indicate the Log_2_ fold change (*x* axis) versus the –Log_10_ *P* value (*y* axis, representing the probability that the gene is differentially expressed). Black dots mark genes with *P* > 0.1 and less than two-fold change, and tinted dots mark genes with fold changes higher than 2. *Ox40l* is marked. **c** mRNA level of *Ox40l* in *Lkb1*^f/f^ and *Cd11c*^Cre^*Lkb1*^f/f^ DCs determined by real-time PCR. **d** Flow cytometric analysis and quantification of the relative mean fluorescence intensity (MFI) of OX40L expression on DCs from the spleen and LNs of *Lkb1*^f/f^ and *Cd11c*^Cre^*Lkb1*^f/f^ mice. **e**–**g** Flow cytometric analysis (**e**), percentage (**f**), and absolute number (**g**) of CFSE-labelled T_reg_ cells (CD4^+^Foxp3^+^, 2×10^5^) sorted from B6 mice after 4 days of co-culture with DCs (1×10^5^) sorted from *Lkb1*^f/f^ and *Cd11c*^Cre^*Lkb1*^f/f^ mice pre-blocked with OX40L neutralizing antibodies or isotype control. **h**–**j** Flow cytometric analysis (**h**), percentage (**i**) and number (**j**) of CFSE-labelled T_reg_ cells (CD4^+^Foxp3^+^, 2×10^6^) sorted from B6 mice transferred together with or without DCs (1×10^6^) sorted from *Lkb1*^f/f^ or *Cd11c*^Cre^*Lkb1*^f/f^ mice pre-blocked with OX40L neutralizing antibodies or isotype control into irradiated NSG mice via tail vein injection for 3 days. The results are presented as the mean ± S.E.M., *P < 0.05, **P < 0.01, ***P < 0.001, by Student's *t*-test (**c**, **d**, **f**, **g**, **i**, **j**). Data are pooled from **c**, **d**, **f**, **g**, **i**, **j** or are representative of at least three **e**, **h** independent experiments with similar results

To elucidate the molecular mechanism by which Lkb1 controls DC function, we profiled the transcriptome of DCs sorted from *Lkb1*^f/f^ and *Cd11c*^Cre^*Lkb1*^f/f^ mice. We found more than 500 transcripts that were significantly (*P* < 0.05) up- or downregulated (1.5-fold or more) in Lkb1-deficient DCs compared with WT DCs and thus constituted the Lkb1-deficient DC-specific transcriptional signature (Supplementary Data 1). Since Lkb1-deficient DCs promoted T_reg_ cell proliferation in a contact-dependent manner, we first screened the significantly altered pathways related to cell adhesion through gene set enrichment analysis (GSEA) and noted that the gene set of cell-cell adhesion was enriched in Lkb1-deficient DCs (Fig. 4a). Among the genes upregulated in Lkb1-deficient DCs are several that have been previously implicated in immune adhesion, including the transcripts of *Ox40l* (*Tnfsf4*)^29–31^, *Cd40*^32^, *Fas*^33^ and *Pdcd1*^34^ (Fig. 4b, Supplementary Data 1). The differential expression of these molecules at the mRNA and protein levels was evaluated by real-time PCR and flow cytometry, respectively. Lkb1 deficiency moderately increased the protein expression of OX40L and CD40 but barely affected that of Fas and PD1 (Fig. 4c, d and Supplementary Fig. 1c). However, recent studies have shown that transgenic mice with constitutive CD11c-specific CD40 signalling had low T_reg_ cell frequencies^35,36^, suggesting that the expansion of T_reg_ cells was unlikely due to CD40 upregulation on Lkb1-deficient DCs. We also examined the expression of OX40L on different subpopulations of DCs and found that OX40L was marginally expressed on pDCs and CD8^+^ cDCs from the spleen and LNs. However, the expression of OX40L on CD11b^+^ DCs from the spleen and LNs was higher in *Cd11c*^Cre^*Lkb1*^f/f^ mice than in *Lkb1*^f/f^ mice (Supplementary Fig. 7a, b). OX40L is a member of the TNF superfamily that has been implicated in DC-T cell interactions^29–31^. The higher expression of OX40 on T_reg_ cells than on naive and activated CD4^+^ T cells (Supplementary Fig. 8a, b) suggests that OX40L-OX40 interactions might preferentially promote T_reg_ cell proliferation.

Pre-blocking OX40L with a neutralizing antibody significantly hampered the increased proliferation and absolute number of T_reg_ cells when co-cultured with Lkb1-deficient DCs but not when co-cultured with WT DCs (Fig. 4e–g). Since the co-stimulatory molecules CD80 and CD86 expressed on DCs have been suggested to be strong stimulators of T_reg_ cell proliferation^37^, we took CD80 and CD86 as positive controls and found that blocking CD80 and/or CD86 on both WT and Lkb1-deficient DCs suppressed T_reg_ proliferation (Supplementary Fig. 9a, b), which is consistent with the comparable expression of CD80 and CD86 on DCs from *Lkb1*^f/f^ and *Cd11c*^Cre^*Lkb1*^f/f^ mice (Supplementary Fig. 1c). The effect of blocking OX40L on DCs from *Cd11c*^Cre^*Lkb1*^f/f^ mice was obvious, indicating that it plays a significant role in enhancing T_reg_ cell expansion.

To confirm this result in vivo, DCs sorted from *Lkb1*^f/f^ or *Cd11c*^Cre^*Lkb1*^f/f^ mice were pre-incubated with anti-OX40L neutralizing antibody or isotype control and then transferred with CFSE-labelled T_reg_ cells from B6 mice into irradiated NSG mice. After 3 days, we detected significantly greater proliferation and absolute cell number of T_reg_ cells after co-transfer with Lkb1-deficient DCs than after co-transfer with WT DCs, and these increases could be mitigated by blocking OX40L (Fig. 4h–j). These results indicate that increased OX40L expression was involved in the superior T_reg_ cell-stimulating effect of Lkb1-deficient DCs. However, when T_con_ cells from B6 mice were co-cultured with WT and Lkb1-deficient DCs, we also observed increased T_eff_ cell (CD44^hi^CD62L^low^) proliferation that could be restrained with anti-OX40L neutralizing antibody (Supplementary Fig. 10a-c). Although Lkb1-deficient DCs have a slightly increased capacity for promoting T_con_ cell survival compared with that of WT DCs, blocking OX40L did not have significant effect on the survival of T_con_ cells (Supplementary Fig. 10d, e). This phenomenon indicates that the upregulated expression of OX40L on Lkb1-deficient DCs also contributes to the increased proportion of polyclonal T_eff_ cells through enhancing proliferation.

### Lkb1 restrains NF-κB signalling to suppress OX40L expression

**Fig. 5:**
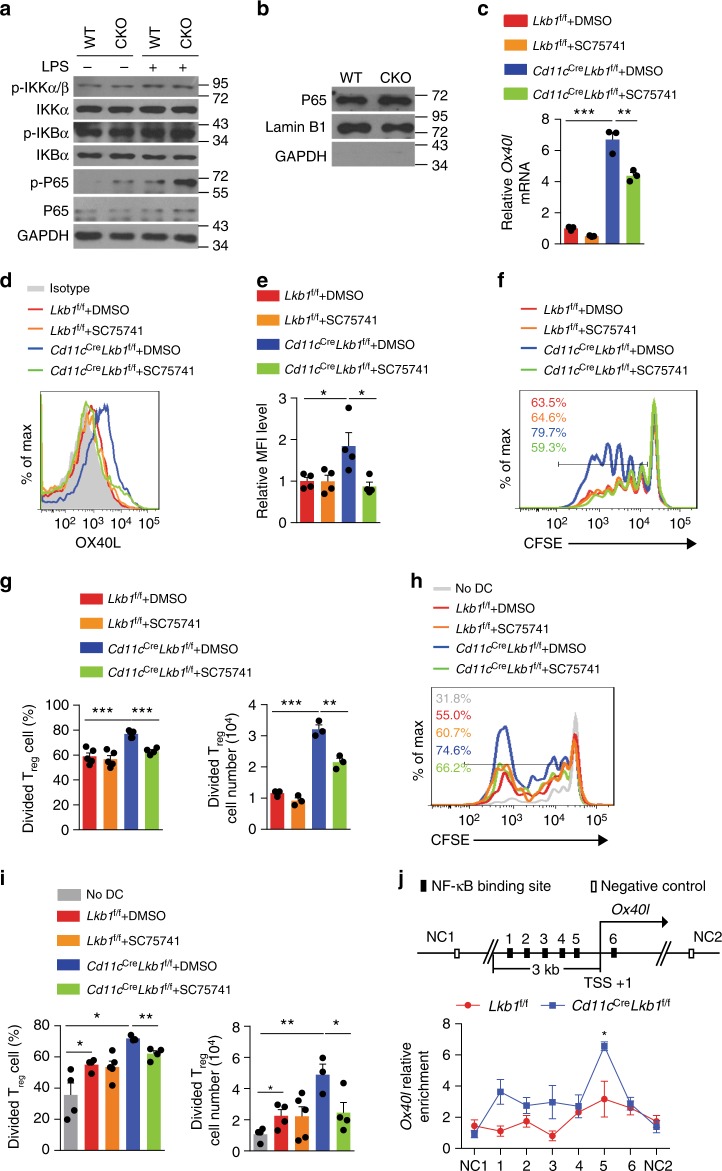
Lkb1 restrains NF-κB signalling to inhibit *Ox40l* expression. **a** Immunoblot analysis of total and phosphorylated p65, IKKα and IκBα expression in WT and Lkb1-deficient DCs treated or untreated with LPS (1μgml^−1^) for 40 min. **b** Immunoblot analysis of p65 protein in the nucleus of splenic DCs from *Lkb1*^f/f^ and *Cd11c*^Cre^*Lkb1*^f/f^ mice. **c** Real-time PCR analysis of the mRNA level of *Ox40l* in splenic DCs from *Lkb1*^f/f^ and *Cd11c*^Cre^*Lkb1*^f/f^ mice treated with or without an NF-κB inhibitor (SC75741) for 3 days. **d**, **e** Flow cytometric analysis (**d**) and quantification (**e**) of relative MFI of OX40L expression on DCs in LNs from *Lkb1*^f/f^ and *Cd11c*^Cre^*Lkb1*^f/f^ mice treated or untreated with an NF-κB inhibitor (SC75741) for 3 days. **f**, **g** Flow cytometric analysis (**f**), percentage and number (**g**) of CFSE-labelled T_reg_ cells co-cultured with DCs from *Lkb1*^f/f^ and *Cd11c*^Cre^*Lkb1*^f/f^ mice treated with or without SC75741 for 4 days. **h**, **i** Flow cytometric analysis (**h**), percentage and number (**i**) of CFSE-labelled T_reg_ cells (CD4^+^Foxp3^+^, 2×10^6^) sorted from B6 mice transferred together with or without DCs (1×10^6^) sorted from *Lkb1*^f/f^ or *Cd11c*^Cre^*Lkb1*^f/f^ mice treated with or without SC75741 into irradiated NSG mice via tail vein injection for 3 days. **j** Relative enrichment of NF-κB p65 to *Ox40l* promoter in *Lkb1*^f/f^ or *Cd11c*^Cre^*Lkb1*^f/f^ DCs treated with LPS (1 μg ml^−1^) for 3 h, determined by ChIP. The results are presented as the mean ± S.E.M., *P < 0.05, **P < 0.01, ***P < 0.001, by Student's *t*-test (**c**, **e**, **g**, **i**). Data are pooled from **e**, **g**, **i**, **j** or are representative of at least three **a**, **b**, **d**, **f**, **h** independent experiment with similar results

We next explored the signalling pathways through which Lkb1 regulates *Ox40l* gene expression. Analysis of the cis-elements of the *Ox40l* gene revealed several conserved NF-κB binding sites (Supplementary Table 1). NF-κB signalling plays critical roles in regulating immune responses^38^. The NF-κB p65 transcription factor is a major component of the NF-κB family, and its phosphorylation marks the activation of the NF-κB pathway. Indeed, the level of phosphorylated NF-κB p65 was higher in Lkb1-deficient DCs than in WT DCs with or without LPS treatment (Fig. 5a and Supplementary Fig. 15a-g). In addition, more p65 was accumulated in the nucleus of Lkb1-deficient DCs (Fig. 5b and Supplementary Fig. 15h-j). Surprisingly, the depletion of Lkb1 did not result in the upstream activation of IKKα/β or IκBα^38^, indicating that Lkb1 deficiency in DCs activated NF-κB signalling distinct from the canonical NF-κB signalling pathway. In addition, the NF-κB inhibitor SC75741 decreased the mRNA and protein levels of OX40L in Lkb1-deficient DCs (Fig. 5c–e). Meanwhile, compared with untreated DCs, Lkb1-deficient DCs pre-treated with the NF-κB inhibitor showed smaller increases in the proliferation and number of T_reg_ cells (Fig. 5f, g). We also confirmed these results in vivo by co-transferring DCs from *Lkb1*^f/f^ and *Cd11c*^Cre^*Lkb1*^f/f^ mice pre-treated with the NF-κB inhibitor with CFSE-labelled T_reg_ cells into irradiated NSG mice (Fig. 5h, i). Furthermore, chromatin immunoprecipitation (ChIP) assays showed more NF-κB p65 binding on the *Ox40l* promotor in Lkb1-deficient DCs than in WT DCs (Fig. 5j), suggesting a direct effect of NF-κB signalling on promoting *Ox40l* transcription. These results suggest that an unusual form of IKKα/β- and IκBα-independent NF-κB activation drove the upregulation of *Ox40l* in *Lkb1*-deficient DCs and that this specifically activated NF-κB signalling promoted T_reg_ cell proliferation at least partially via OX40L.

SIRT1 (sirtuin-1) has been found to interact with the Lkb1/AMPK complex and inhibit NF-κB signalling to regulate innate immunity defences^39,40^. To investigate whether SIRT1 was involved in Lkb1- and LPS-mediated signalling in DCs, we examined the protein level of SIRT1 in Lkb1-deficient DCs and DCs from LPS-treated mice. The total protein level of SIRT1 was diminished in DCs from LPS-treated mice but not in Lkb1-deficient DCs (Supplementary Figs. 11a and 15o, p). Since SIRT1 negatively regulates NF-κB p65 activation^38,39^, the downregulation of SIRT1 may contribute to the activation of NF-κB p65 in DCs from LPS-treated mice. However, SIRT1 is not reduced in Lkb1-deficient DCs, suggesting that Lkb1 loss provokes NF-κB hyperactivation independent of SIRT1. These results indicate that SIRT1 might be involved in the activation process of DCs from LPS-treated mice rather than mediating the Lkb1 effect in DCs. In addition, SIRT1 has been found to interact with the Lkb1/AMPK complex to regulate the downstream signalling^39,41^. However, in our study, Lkb1 controlled DC function independent of AMPK. These results collectively suggest that Lkb1 function in DCs is possibly independent of SIRT1.

### LPS induces T_reg_ cell expansion via depleting Lkb1 in DCs

**Fig. 6:**
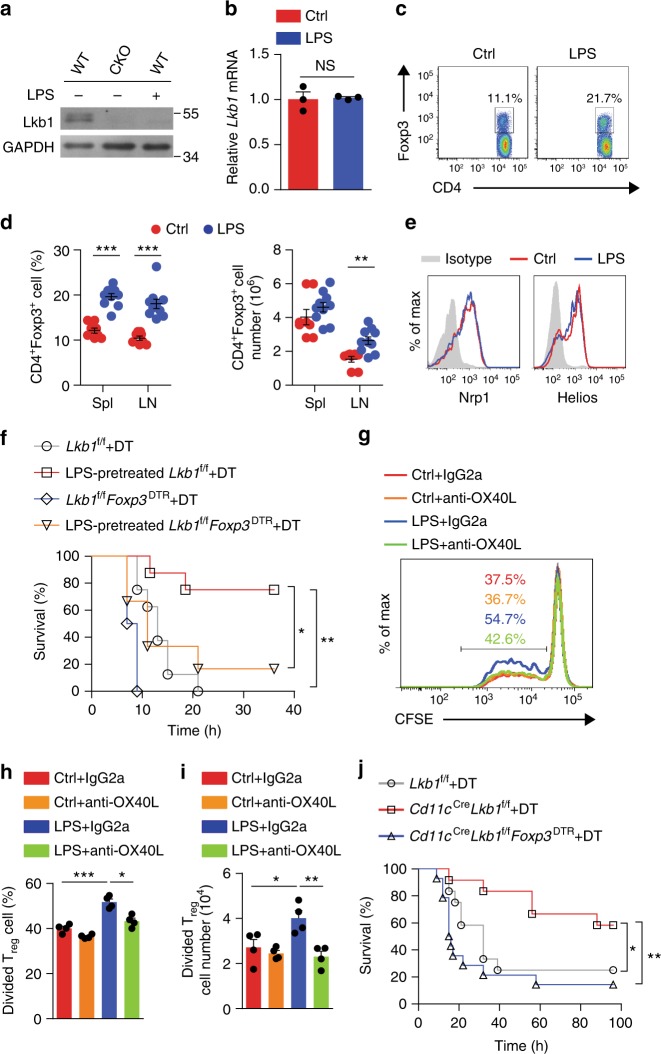
LPS induces T_reg_ cell expansion and suppression through depleting Lkb1 in DCs. **a** Lkb1 protein in splenic DCs from *Lkb1*^f/f^ (WT) and *Cd11c*^Cre^*Lkb1*^f/f^ (CKO) mice treated or untreated with LPS (1.5 mg kg^−1^) overnight. **b** mRNA level of *Lkb1* in splenic DCs from mice with or without LPS treatment. **c** Representative T_reg_ cell frequencies among splenic CD4^+^ T cells from mice treated with or without LPS 5 days in advance. **d** Quantification of the percentage and absolute number of T_reg_ cells in the spleen and LNs of mice treated with or without LPS 5 days in advance (Ctrl, *n*=8; LPS, *n*=10). **e** Expression of Nrp1 and Helios in splenic T_reg_ cells from mice with or without LPS treatment 5 days in advance. **f** The survival curve of *Lkb1*^f/f^ (*n*=8 each group) and *Lkb1*^f/f^*Foxp3*^DTR^ mice (*n*=6 each group) pre-treated with or without LPS (5 days in advance), treated with DT (50 μg kg^−1^, 2 days in advance), and then challenged with a lethal dose of LPS (30 mg kg^−1^). **g**–**i** Flow cytometric analysis (**g**), percentage (**h**) and number (**i**) of CFSE-labelled T_reg_ cells sorted from B6 mice after 4 days of co-culture with DCs sorted from C57 mice with or without LPS (1.5 mg kg^−1^) treatment pre-blocked with OX40L neutralizing antibodies or isotype control. **j** The survival curve of *Lkb1*^f/f^ (*n* = 12), *Cd11c*^Cre^*Lkb1*^f/f^ (*n* = 13), and *Cd11c*^Cre^*Lkb1*^f/f^*Foxp3*^DTR^ (*n* = 14) mice treated with DT and then challenged with a lethal dose of LPS. Each symbol (**d**) indicates an individual mouse; the results are presented as the mean ± S.E.M., **P < 0.01, ***P < 0.001; NS not significant, by Student's *t*-test (**b**, **d**, **h, i**). Log-rank survival curve analysis was used (**f**, **j**). Data are pooled from **b**, **d** or are representative of **a**, **c**, **e**, **g** three independent experiments

The above results show the extreme enlargement of the T_reg_ cell compartment in mice with the *Lkb1* gene conditionally deleted in DCs. Considering the abundance of evidence demonstrating increased frequencies of T_reg_ cells during certain immune responses, including those to bacterial infection^42–45^, we next sought to determine whether the expression of Lkb1 would be decreased in DCs to contribute to the augmentation of T_reg_ cells under these conditions. We intraperitoneally treated mice with LPS, a Toll-like receptor (TLR) agonist derived from gram-negative bacteria, which is capable of triggering strong immune activation. Interestingly, we found that the Lkb1 protein level was greatly reduced in DCs (Fig. 6a and Supplementary 15k, l) but not in T and B lineage cells (Supplementary Fig. 12a and 15q-t). Despite a significant reduction in the Lkb1 protein level in DCs, no obvious change in the *Lkb1* mRNA level was observed under LPS stimulation (Fig. 6b), suggesting that LPS might deplete Lkb1 protein via a post-transcriptional mechanism.

As expected, we observed elevated percentages and absolute numbers of T_reg_ cells in mice under LPS stimulation (Fig. 6c, d). The majority of these T_reg_ cells expressed high levels of Nrp1 and Helios (Fig. 6e), indicating that they were mostly nT_reg_ cells. To determine the impact of the LPS-induced increase in T_reg_ cells on the prognosis of an immune response, we pre-treated mice with or without a low dose of LPS before a lethal dose of LPS. The LPS pre-treatment protected *Lkb1*^f/f^ mice from the lethal LPS challenge, but this protective effect was significantly abolished in *Lkb1*^f/f^*Foxp3*^DTR^ mice treated with DT (Fig. 6f). These findings indicate the critical role of increased T_reg_ cell populations in mediating protection against LPS-mediated inflammatory injury.

As LPS could affect the phenotype and function of multiple cell types, to confirm that the expansion of the T_reg_ cell compartment was directly caused by DCs in LPS-treated mice, we sorted DCs from mice treated with or without LPS, incubated them with OX40L neutralizing antibody or isotype control, and co-cultured them with CFSE-labelled T_reg_ cells for 4 days. Flow cytometry analysis showed that LPS-modified DCs promoted stronger T_reg_ cell proliferation than did control DCs and that blocking with anti-OX40L antibody could restrain the proliferation of T_reg_ cells in the LPS group, but not in the control group (Fig. 6g–i). These results suggest that the T_reg_ cell expansion in the mice was directly caused by LPS-programmed DCs and that this effect was at least partially mediated by OX40L. To evaluate whether the Lkb1 protein reduction in DCs alone would contribute to the protective effect under LPS stimulation, we administered a lethal dose of LPS to *Lkb1*^f/f^, *Cd11c*^Cre^*Lkb1*^f/f^ and *Cd11c*^Cre^*Lkb1*^f/f^*Foxp3*^DTR^ mice treated with DT and analysed the survival rate. The survival rate of the *Cd11c*^Cre^*Lkb1*^f/f^ mice was significantly higher than that of the other two groups of mice (Fig. 6j), but the *Cd11c*^Cre^*Lkb1*^f/f^*Foxp3*^DTR^ mice treated with DT, which had reduced T_reg_ cell populations, displayed significantly reduced survival. However, there was no statistically significant difference in the survival rate between the *Lkb1*^f/f^*Foxp3*^DTR^ and *Cd11c*^Cre^*Lkb1*^f/f^*Foxp3*^DTR^ mice treated with DT when challenged with a lethal LPS dose (Supplementary Fig. 13a). Thus, the LPS-induced Lkb1 depletion in DCs was directly involved in promoting T_reg_ cell expansion to enhance immunosuppression. To further explore how Lkb1 deletion-induced T_reg_ expansion would protect the mice from LPS toxicity, we examined the pro-inflammatory cytokine levels and T cell effector function of *Lkb1*^f/f^ and *Cd11c*^Cre^*Lkb1*^f/f^ mice in response to LPS. The mRNA levels of *Tnf* and *Ifnγ* were lower in the lungs of the *Cd11c*^Cre^*Lkb1*^f/f^ mice than in those of the *Lkb1*^f/f^ mice (Supplementary Fig. 14a). We also examined the production of Th-associated cytokines by T cells in *Lkb1*^f/f^ and *Cd11c*^Cre^*Lkb1*^f/f^ mice and found lower IFN-γ production in T cells from the lungs and spleen of *Cd11c*^Cre^*Lkb1*^f/f^ mice (Supplementary Fig. 14b, c). These results indicate that the reduced production of TNF and IFN-γ in the lung tissue and the decreased numbers of IFN-γ-producing T cells might be part of the mechanisms contributing to the alleviated LPS toxicity.

### *E. coli* induces T_reg_ cell expansion by depleting Lkb1 in DCs

**Fig. 7:**
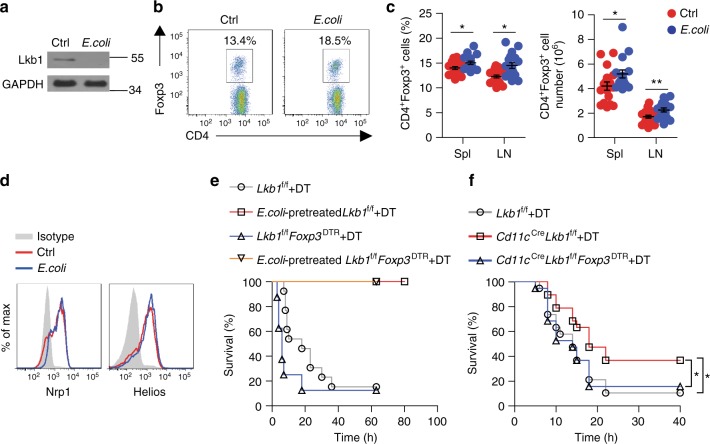
*E. coli* infection induces T_reg_ cell expansion and suppression through depleting Lkb1 in DCs. **a** Immunoblot analysis of Lkb1 protein in DCs from C57 mice challenged with or without *E. coli* (1×10^7^) via intraperitoneal injection overnight. **b** Flow cytometric analysis of splenic T_reg_ cells from C57 mice treated with or without *E. coli* (1×10^7^) 5 days in advance. **c** Percentage and absolute number of T_reg_ cells in the spleen and LNs of C57 mice treated with or without *E. coli* (1×10^7^) 5 days in advance (Ctrl, *n* = 18; *E. coli*, *n* = 17). **d** Flow cytometric analysis of Nrp1 and Helios expression on splenic T_reg_ cells from C57 mice challenged with or without *E. coli* (1×10^7^) 5 days in advance. **e** The survival curve of *Lkb1*^f/f^ (*n* = 13 each group) and *Lkb1*^f/f^*Foxp3*^DTR^ mice (*n* = 8 each group) pre-treated with or without *E. coli* (1×10^7^, 5 days in advance), treated with DT (50 μg kg^−1^, 2 days in advance), and then challenged with a lethal dose of *E. coli* (1×10^8^). **f** The survival curve of *Lkb1*^f/f^ mice, *Cd11c*^Cre^*Lkb1*^f/f^ mice, and *Cd11c*^Cre^*Lkb1*^f/f^*Foxp3*^DTR^ mice treated with DT (50 μg kg^−1^, 2 days in advance) and challenged with a lethal dose of *E. coli* (1×10^8^) administered by intraperitoneal injection (*n* = 19 each group). Each symbol (**c**) indicates an individual mouse; the results are presented as the mean ± S.E.M., *P < 0.05, **P < 0.01, by Student's *t*-test (**c**). Log-rank survival curve analysis was used (**e**, **f**). Data are pooled from **c**, **f** or are representative of three **a**, **b**, **d**, **e** independent experiments with similar results

Because LPS is a major pathogenic factor derived from gram-negative bacteria, we further tested whether infection with the gram-negative bacteria *E. coli* could recapitulate the effect of LPS. Consistently, the decrease in Lkb1 protein was also observed in DCs from mice with an intraperitoneal *E. coli* infection (Fig. 7a and Supplementary Fig. 15m, n), along with an increased frequency and absolute number of T_reg_ cells (Fig. 7b, c). These T_reg_ cells were mostly nT_reg_ cells given their high expression of Nrp1 and Helios (Fig. 7d). *E. coli* pre-treatment protected mice from secondary lethal *E. coli* challenge. Surprisingly, reducing the T_reg_ cell population with DT did not abolish the protective effect of the *E. coli* pre-treatment (Fig. 7e), indicating that mechanisms other than T_reg_ cells predominately mediated the protection from a secondary infection. As gram-negative bacteria, *E. coli* contain more complex virulence factors and have been shown to induce immune tolerance by multiple mechanisms^46^, which might override the protective effect of the bacteria pre-treatment-induced increase in the T_reg_ cell population. To further determine whether Lkb1 depletion in DCs alone is sufficient to mediate tolerance to an acute primary lethal *E. coli* challenge, we administered lethal amounts of *E. coli* to *Lkb1*^f/f^, *Cd11c*^Cre^*Lkb1*^f/f^, and *Cd11c*^Cre^*Lkb1*^f/f^*Foxp3*^DTR^ mice treated with DT. The *Cd11c*^Cre^*Lkb1*^f/f^ mice showed a significantly higher survival rate than the *Lkb1*^f/f^ mice upon lethal *E. coli* challenge, and reducing the T_reg_ cell population in the *Cd11c*^Cre^*Lkb1*^f/f^*Foxp3*^DTR^ mice through DT treatment resulted in poor survival (Fig. 7f), indicating that conditional knockout of the *Lkb1* gene in DCs increases the T_reg_ cell number and the tolerance of the host to primary bacterial infections. Finally, *Lkb1*^f/f^*Foxp3*^DTR^ and *Cd11c*^Cre^*Lkb1*^f/f^*Foxp3*^DTR^ mice treated with DT showed comparable survival rates when challenged with a lethal dose of *E. coli* (Supplementary Fig. 13b). These results indicate that the Lkb1 depletion-induced expansion of the T_reg_ cell population promotes tolerance to primary bacterial infections but is dispensable for establishing tolerance to secondary infections.

### Lkb1 discriminates regulatory from inflammatory programmes

**Fig. 8:**
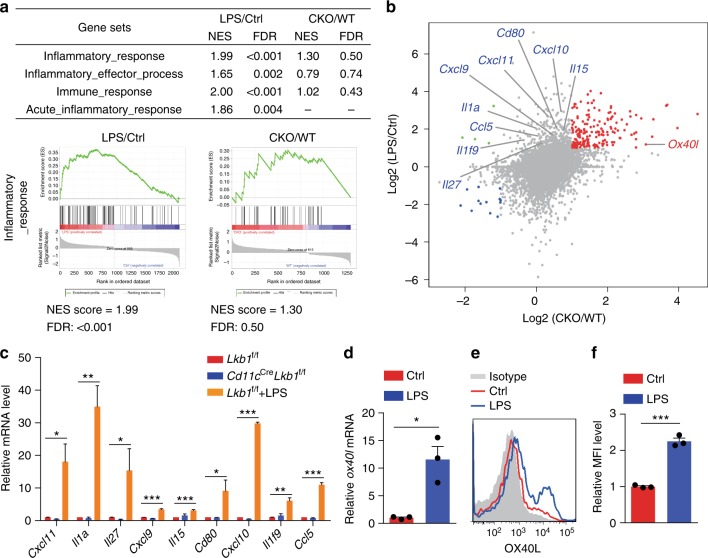
Lkb1 discriminates regulatory from inflammatory transcriptional programmes. **a** GSEA of transcriptional profiles of DCs from mice treated with LPS compared with that of *Cd11c*^Cre^*Lkb1*^f/f^ mice. The upper part shows the list of gene sets related to inflammation; the lower part shows a representative plot of the gene set; –, no enrichment. **b** Scatter plot of Log_2_ counts of the genes expressed in splenic DCs from *Lkb1*^f/f^ mice treated with LPS (*n* = 1) compared with those from *Cd11c*^Cre^*Lkb1*^f/f^ mice (*n* = 3). Grey dots mark genes with less than two-fold change in any group. Red and blue dots mark the positively correlated genes with more than two-fold change from the two groups, while negatively correlated genes are indicated in green. The names of pro-inflammatory genes only upregulated in LPS-treated DCs are indicated in blue characters, and *Ox40l*, upregulated in both groups, is indicated in red characters. **c** mRNA levels of *Cxcl11, Il1a, Il27, Cxcl9, Il15, Cd80, Il1f9, Cxcl10* and *Ccl5* in splenic DCs from *Lkb1*^f/f^ mice, *Lkb1*^f/f^ mice treated with LPS, and *Cd11c*^Cre^*Lkb1*^f/f^ mice. **d** mRNA level of *Ox40l* in DCs sorted from mice treated with or without LPS. **e**, **f** Flow cytometric analysis (**e**) and quantification of relative MFI (**f**) of OX40L protein expression on splenic DCs from mice treated with or without LPS (1.5 mg kg^−1^). The results are presented as the mean ± S.E.M., *P < 0.05, **P < 0.01, ***P < 0.001, by Student's *t*-test (**c**, **d**, **f**). Data are pooled from **c, d, f** or are representative of **e** at least two independent experiments with similar results

While LPS is a well-known pathogenic factor that acts on DCs to promote inflammation, here, we show that LPS also depletes Lkb1 in DCs to induce T_reg_ cell expansion and immunosuppression. To understand the molecular basis that differentiates the regulatory and pro-inflammatory functions, we further profiled the transcriptome of DCs sorted from mice treated with or without LPS for comparison with the aforementioned transcriptional signature of Lkb1-deficient DCs (Supplementary Data 1 and 2). GSEA showed that pathways related to inflammatory responses, inflammatory effector processes, immune responses and acute immune responses were significantly upregulated in DCs from LPS-treated mice, while no significant enrichment in these gene sets appeared in Lkb1-deficient DCs (Fig. 8a). A substantial proportion of the upregulated transcripts in DCs from LPS-treated mice were not significantly altered in Lkb1-deficient DCs, including genes encoding the pro-inflammatory cytokines *Il1α*, *Il27*, *Il15* and *Il1f9*, the costimulation molecule *Cd80*, and the chemokines *Cxcl11*, *Cxcl9*, *Cxcl10* and *Ccl5*, which was confirmed by real-time PCR (Fig. 8b, c and Supplementary Data 2). However, *Ox40l*, the upregulated transcript in Lkb1-deficient DCs, was also increased in DCs from LPS-treated mice, and real-time PCR and flow cytometry further confirmed that LPS could upregulate OX40L expression in DCs (Fig. 8d–f). These results demonstrate that Lkb1 operated as a “regulatory switch” to discriminate the regulatory from the pro-inflammatory transcriptional programme in DCs.

## Discussion

Along with tolerogenic DCs that are thought to be associated with developmental immaturity^45,47,48^, the concept of “regulatory DCs”, which are proposed to evolve under certain immune conditions and exhibit a superior immunosuppressive capacity, has been considered for decades^10,45^, but the underlying mechanism that governs the specification of this distinctive lineage is still elusive. In this study, we demonstrate that a deficiency in Lkb1, achieved through either artificial gene deletion or bacteria/LPS-induced protein depletion, establishes the regulatory function of DCs to augment T_reg_ cell expansion under either steady-state or inflammatory conditions. These findings indicate a mechanism underlying this regulatory functional specification.

An increasing body of evidence suggests that bacterial infection not only induces the activation of effector cells to eliminate pathogens but also elicits immunosuppression that involves iT_reg_ cell de novo generation^1,45,49^. In our study, judging from the positive expression of Nrp1 and Helios, nT_reg_ but not iT_reg_ cells are major components of the enlarged T_reg_ cell pool in mice intraperitoneally challenged with bacteria or LPS. We showed that bacteria and LPS pre-treatment provided protection against a lethal secondary infection. LPS pre-treatment-induced Lkb1 depletion promoted T_reg_ expansion to protect mice from a secondary lethal challenge. However, *E. coli* pre-treatment-induced protection from the secondary lethal infection was largely independent of T_reg_ expansion, indicating that other tolerance mechanisms might exist, which may override the effect of T_reg_ cell expansion during bacterial infection. Nonetheless, functional genomic analysis revealed that the Lkb1 deficiency established a regulatory transcriptional programme in DCs without interfering with the pro-inflammatory transcriptional programme, thus providing a unique molecular basis for bacterial infection-induced tolerance.

Despite the well-recognized role of OX40L–OX40 interactions in promoting effector T cell responses^29,30^, the impact of OX40L–OX40 interactions on T_reg_ cells remains controversial. Some reports have suggested that OX40 favours T_reg_ cell suppression via increasing expansion^27,50^, while others have demonstrated that OX40 represss T_reg_ cell suppressive function due to T_reg_ cell exhaustion and a lack of IL-2^31^. Our study defines the role of OX40L–OX40 interactions in promoting T_reg_ cell proliferation driven by Lkb1-deficient DCs. Although the upregulation of OX40L on Lkb1-deficient DCs was moderate, it had a significant impact on T_reg_ cell proliferation, as blocking OX40L on Lkb1-deficient DCs reduced T_reg_ cell proliferation to an extent similar to that of blocking both CD80 and CD86, which are known as strong stimulators of T_reg_ cell proliferation^37^. However, blocking OX40L on Lkb1-deficient DCs could only partially rescue the enhanced proliferation of T_reg_ cells, suggesting that other molecules might also contribute to T_reg_ cell proliferation. Indeed, Lkb1 also suppressed the expression of other cell membrane molecules, including semaphorins, which are involved in cell adhesion and communication^51,52^, and CCL22, which is thought to promote T_reg_ cell chemotaxis^53,54^. The impact of these molecules on T_reg_ cells within the complex immune microenvironment cannot be excluded. Therefore, we propose that Lkb1 might control a number of genes in DCs to govern the magnitude of T_reg_ cell expansion in vivo.

We found that NF-κB p65 activation induced *Ox40l* expression in Lkb1-deficient DCs and that the inhibition of NF-κB signalling in Lkb1-deficient DCs reduced the proliferation of T_reg_ cells, suggesting that NF-κB activation in Lkb1-deficient DCs promotes T_reg_ cell proliferation at least via the upregulation of OX40L. NF-κB is a well-known mediator of TLR-triggered pro-inflammatory immune responses^38^. Therefore, it was surprising that Lkb1 deficiency-triggered p65 activation promoted the regulatory but not the pro-inflammatory function of DCs. Indeed, the conditional deletion of Lkb1 did not lead to the activation of IKKα/β or the degradation of IκBα. This unusual Lkb1 regulation of NF-κB signalling is in contrast with the results of previous studies showing that Lkb1 regulates IKKα/β activation in other immune cell types;^18,19^ thus, this might be a unique mechanism for discriminating the regulatory programme from the pro-inflammatory programme in DCs. In addition to OX40L, we cannot exclude the possibility that Lkb1-controlled NF-κB signalling might regulate other molecules to affect T_reg_ cell homeostasis, which awaits future clarification.

Lkb1 also plays intrinsic roles in T_reg_ cells. Our previous work demonstrates that Lkb1 promotes T_reg_ cell Foxp3 expression and suppressive function^18^. In addition, Lkb1 was recently shown to programme T_reg_ cell metabolic and functional fitness^55^. Intriguingly, here, we show that Lkb1 operates in DCs to negatively regulate T_reg_ cell expansion and immunosuppression. Furthermore, LPS treatment selectively depleted Lkb1 in DCs but not in T_reg_ cells to potentiate immunosuppression. Surprisingly, the conditional deletion of Lkb1 in monocytes/macrophages, which share certain functions (e.g., antigen presentation, inflammatory mediator production) with DCs, did not lead to T_reg_ cell expansion. Together, these results highlight an environment- and cell type-conditioned function of Lkb1 in the orchestration of immunity versus tolerance.

In conclusion, these findings indicate that the abundance and immunosuppression strength of T_reg_ cells are dynamically governed, in both a feedforward and feedback manner, by the Lkb1 “regulatory switch” in DCs to maintain immune equilibrium. Our study also revises the concept of “regulatory DCs” by providing evidence supporting a unifying model of regulatory and inflammatory programmes that can co-evolve but are separately instructed by different signals in the same DCs during maturation/activation. These findings provide insight into the sophisticated DC-T_reg_ cell interactions that are fundamental for controlling immune equilibrium. Given that dysregulated T_reg_ cell pools are involved in the pathogenesis of various cancerous and autoimmune diseases, our work may provide therapeutic targets for the clinical treatment of these T_reg_ cell-related immune diseases.

## Methods

### Mice

All animals were maintained in specific pathogen-free barrier facilities and used in accordance with protocols approved by the Institutional Animal Care and User Committee at the Institute of Hematology, Chinese Academy of Medical Sciences. C57BL/6, *Lkb1*^f/f^, *AMPKα1*^f/f^, *AMPKα2*^f/f^, *Cd11c*^Cre^, *Foxp3*^YFP-Cre^, *LysM*^Cre^, *Foxp3*^DTR^ and NOD/scid IL2Rg^null^ (NSG) mice were purchased from Jackson Laboratories. All mice had been backcrossed with C57BL/6 mice for at least seven generations. *Lkb1*^f/f^, *AMPKα1*^f/f^ and *AMPKα2*^f/f^ mice were crossed with *Cd11c*^Cre^, *LysM*^Cre^ to generate *Cd11c*^Cre^*Lkb1*^f/f^, *Cd11c*^Cre^*AMPKα1*^f/f^*AMPKα2*^f/f^, and *LysM*^Cre^*Lkb1*^f/f^ mice, respectively. *Cd11c*^Cre^*Lkb1*^f/f^ mice were crossed with *Foxp3*^DTR^ mice to generate *Cd11c*^Cre^*Lkb1*^f/f^*Foxp3*^DTR^ mice. All mice were used when 6–8 weeks old unless otherwise noted. The sample size was selected to maximize the chance of uncovering a mean difference with statistical significance. No statistical methods were used to predetermine the sample size. The experiments were not randomized, and the investigators were not blinded to group allocation during the experiments or outcome assessments.

### Cell purification and flow cytometry

For the analysis of cell surface markers, single-cell suspensions were prepared from spleen and LN samples for staining with APC-Cy7-anti-CD4 (100413, Biolegend), APC-Cy7-anti-CD45.1 (110715, Biolegend), APC-anti-CD45.2 (109813, Biolegend), APC-anti-CD4 (100411, Biolegend), APC-anti-MHC II (107613, Biolegend), APC-anti-CD62L (104411, Biolegend), APC-anti-CD304 (neuropilin-1) (145205, Biolegend), PerCP-anti-CD80 (104721, Biolegend), PerCP-anti-CD45.2 (109825, Biolegend), PerCP-anti-CD44 (103035, Biolegend), PerCP-anti-CD8α (100731, Biolegend), FITC-anti-CD62L (104405, Biolegend), FITC-anti-CD279 (PD1) (135213, Biolegend), FITC-anti-CD45.1 (110705, Biolegend), PE-Cy7-anti-CD11c (117317, Biolegend), PE-Cy7-CD45.1 (110729, Biolegend), PE-Cy7-CD86 (105013, Biolegend), PE-Cy7-anti-CD8α (100721, Biolegend), PE-Cy7-anti-CD45.2 (109829, Biolegend), PE-Cy7-anti-CD278 (ICOS) (25–9942–80, eBioscience), PE-anti-CD25 (101903, Biolegend), PE-anti-CD252 (*Ox40l*) (12-5905-81, eBioscience), PE-anti-CD40 (124609, Biolegend), PE-anti-CD95 (Fas) (152607, Biolegend), and PE-anti-CD11c (117307, Biolegend). These antibodies were obtained from eBioscience or Biolegend. CD4^+^ T cells, CD8^+^ T and CD11c^+^ DCs were purified with Dynabeads Untouched Mouse CD4 and CD8 Cell Kits (11416D, 11417D, Invitrogen) and CD11c MicroBeads (130-108-338, Miltenyi Biotec), respectively. The indicated T_reg_ cell populations were sorted from purified CD4^+^ T cells using a FACSAria III system (BD Biosciences), and the sorted populations were >98% pure unless otherwise specified. Intracellular staining with PE-Foxp3 (12-5773-82, eBioscience), APC-anti-Helios (137221, Biolegend), APC-anti-Ki67 (652405, Biolegend), and antibodies to cytokines, including APC-anti-IL-17 (506915, Biolegend), FITC-anti-IL-2 (503805, Biolegend), and PE-anti-IFN-γ (505807, Biolegend) (spleen cells were stimulated with phorbol myristate acetate (PMA, 50 ng ml^-1^) and ionomycin (500 ng ml^−1^) (Sigma-Aldrich) for 4 h before analysis of the cytokine expression in the indicated populations), were performed with Foxp3 staining kits (72-5775-40, eBioscience). Cell surface staining was mostly performed at 4 °C for 30 min. OX40L on splenic DCs was stained at room temperature for 2–3 h. Flow cytometry data were acquired on an LSR II, LSRFortessa (BD Biosciences) or FACSCanto II (BD Biosciences) system and analysed with FlowJo software (Tree Star). Gating strategies are described in Supplemental Table 3.

### T_reg_ cell proliferation

CD4^+^CD25^+^ T_reg_ cells sorted from B6 mice were confirmed to consist of more than 95% of Foxp3^+^ cells and labelled using CFSE Cell Proliferation Kits (Invitrogen) for 8 min. at room temperature and then washed twice with phosphate-buffered saline (PBS). For the *in vitro* study, CFSE-labelled T_reg_ cells were co-cultured with CD11c^+^ cells purified from *Cd11c*^Cre^*Lkb1*^f/f^ and *Lkb1*^f/f^ mice in the presence or absence of recombinant murine IL-2 (100 ng/ml, Biolegend) for 4 days. For the in vivo study, to exclude the influence of DCs from the host, we used NSG mice as recipient mice, whose DCs are defective; 2 × 10^6^ CFSE-labelled T_reg_ cells were transferred together with 1 × 10^6^ DCs purified from *Lkb1*^f/f^ or *Cd11c*^Cre^*Lkb1*^f/f^ mice into sublethally (2 Gy) irradiated NSG mice by tail vein injection. After 3 days, the spleen was removed, and the proliferation of CD4^+^Foxp3^+^ T_reg_ cells was analysed by flow cytometry. In some experiments, DCs were treated with OX40L (R&D, MAB1236 and Bioxcell, BE0033), CD80 (R&D, AF740), and CD86 (R&D, AF-441-NA) neutralizing antibodies or their isotype controls at a concentration of 30 μg/ml at 4 °C for 1.5 h. In some experiments, DCs are sorted from *Lkb1*^f/f^ or *Cd11c*^Cre^*Lkb1*^f/f^ mice treated with or without NF-κB inhibitor SC75741(Selleck, S7273). Mixed BM chimaera mouse models were established by co-transferring equal numbers of total BM cells (1 × 10^7^) from *Lkb1*^f/f^ (CD45.1^+^CD45.2^+^) and *Cd11c*^Cre^*Lkb1*^f/f^ (CD45.2^+^) mice together into lethally irradiated (8 Gy) B6 (CD45.1^+^) mice, followed by reconstitution for at least 6 weeks.

### T_reg_ cell suppression

CD4^+^CD25^-^CD44^lo^CD62L^hi^ naïve T (T_n_) cells sorted from CD45.1^+^ mice were labelled with CFSE and used as responder cells (T_resp_). T_resp_ cells (5 × 10^4^) were cultured for 3 days with DCs (1 × 10^5^) in the presence or absence of the indicated numbers of CD4^+^CD25^**+**^ T_reg_ cells sorted from *Lkb1*^f/f^ or *Cd11c*^Cre^*Lkb1*^f/f^ mice.

### Apoptosis detection

Annexin V and PI staining was performed using an apoptosis detection kit (Biolegend) according to the manufacturer’s instruction to determine the apoptosis of T_reg_ cells in the spleen from *Lkb1*^f/f^ and *Cd11c*^Cre^*Lkb1*^f/f^ mice.

### T cell proliferation and activation

CD4^+^ and CD8^+^ T cells were sorted from OT-II and OT-I transgenic mice, labelled with CFSE, and then co-cultured with *Cd11c*^Cre^*Lkb1*^f/f^ and *Lkb1*^f/f^ DCs loaded with the respective antigen peptide (OVA _323-339_ and OVA_257–264_, Sigma-Aldrich) for 4 days. For the in vivo study, *Lkb1*^f/f^, *Cd11c*^Cre^*Lkb1*^f/f^ and *Cd11c*^Cre^*Lkb1*^f/f^*Foxp3*^DTR^ mice treated were with diphtheria toxin (DT) for 2 days; then, CFSE-labelled CD4^+^ T cells were transferred into these mice via tail vein injection. After 1 day, the mice were challenged by a subcutaneous injection of 20μg of OVA protein (A5503, Sigma-Aldrich) with 100 μl of Complete Freund's adjuvant (CFA). In addition, 3 days later, the LNs were removed and the cell populations were analysed by flow cytometry.

### Immunoblot analysis

DCs were sorted from *Lkb1*^f/f^ and *Cd11c*^Cre^*Lkb1*^f/f^ mice and treated as indicated, and then the cells were lysed with RIPA buffer or nuclear/cytosol fractionation reagent (Bio Vision) supplemented with protease and phosphatase inhibitors. The protein concentration in the extract was measured by BCA assay. The same amount of protein for each sample was separated by sodium dodecyl sulfate–polyacrylamide gel electrophoresis (SDS-PAGE), transferred by electroblotting and membranes blocked in 5% milk/Tris buffered saline with Tween 20 (TBS-T) for 1 h. Membranes were incubated with appropriate antibodies overnight at 4 °C, then exposed to secondary antibodies for 2 h at room temperature and developed using ECL Western Blotting Substrate. Western blot was carried out with antibodies against Lkb1 (1:500), phospho-IKKα/β (Ser176/180, 1:500), phospho-IκBα (Ser32, 1:500), phospho-NF-κB p65 (Ser536, 1:500), IKKα (1:500), IκBα (1:500), NF-κB p65 (1:500) and GAPDH (1:1000). These antibodies were purchased from Cell signalling Technology, USA. Uncropped scans of all the blots were provided in the Supplementary Figure 15.

### Microarray and quantitative real-time PCR

DCs were sorted from the spleen of *Lkb1*^f/f^ mice, LPS-treated mice, and *Cd11c*^Cre^*Lkb1*^f/f^ mice for RNA extraction with TRIzol reagent (Invitrogen). Total RNA was reverse-transcribed, amplified, labelled, and hybridized to Mouse Genome 2.0 arrays (Affymetrix). The microarray data sets were analysed using Agilent GeneSpring GS 11 software and GSEA. RNA from different samples was obtained in the same manner as for the microarray analysis, and real-time PCR was performed with SYBR Green PCR Master Mix (ABI). The sequences of the primer pairs used are listed in Supplementary Table 2.

### ELISA

The concentrations of IgG in the serum of *Lkb1*^f/f^ and *Cd11c*^Cre^*Lkb1*^f/f^ mice were determined by sandwich ELISA (Biolegend), according to the manufacturer's instructions.

### Chromatin immunoprecipitation (ChIP)

ChIP assays were performed using ChIP kits (Active Motif) according to the manufacturer’s protocols. Precipitated DNA and input DNA were assessed by real-time PCR using the primers listed in Supplementary Table 2.

### EAE model

*Lkb1*^f/f^ and *Cd11c*^Cre^*Lkb1*^f/f^ female mice (8–10 weeks old) were immunized subcutaneously (s.c.) with MOG35–55 emulsified in CFA and were administered pertussis toxin (PT) intraperitoneally (i.p.) after 0 and 2 days. In our experiments, the EAE symptoms usually started between days 14 and 28. Disease symptoms were regularly monitored and scored as follows: 0: no clinical signs; 1: flaccid tail; 2: hind limb weakness or abnormal gait; 3: complete hind limb paralysis; 4: complete hind limb paralysis + forelimb weakness or paralysis; 5: moribund or deceased (0–5 graduations with 0.5 for intermediate scores).

### Injection of LPS and bacteria

LPS (Sigma-Aldrich) or *E. coli* provided by Prof. Yuanfu Xu (Chinese Academy of Medical Sciences) in 200 μl of PBS was administered by tail vein or intraperitoneal injection. Bacteria were stored in 20% glycerol at −80 °C. For expansion, bacteria were cultured on LB agar through plated streaking overnight. One colony of bacteria was selected, suspended in 4 ml of LB and incubated for 12 h (200 revolutions/min, 37 °C). The suspension was then centrifuged at 8000 rpm for 5 min. The pellet was washed twice with PBS and then resuspended in PBS. The determined concentration of the suspension was adjusted to the desired dose using PBS.

### Statistics

An unpaired two-tailed Student's *t*-test (for the comparison of two groups) or two-way ANOVA (for the comparison of more than two groups) were performed using Prism (GraphPad) to calculate the statistical significance of differences in the mean values as indicated by the *P* value. *P* values < 0.05 were considered statistically significant. *P < 0.05; **P < 0.01; ***P < 0.001.

## Electronic supplementary material

Supplementary Information

Description of Additional Supplementary Files

Supplementary Data 1

Supplementary Data 2

Reporting Summary

## Data Availability

The data that support the findings of this study are available from the corresponding author on reasonable request. The microarray data that support the findings of this study have been deposited in the Gene Expression Omnibus under accession number GSE117286. A reporting summary for this Article is available as a Supplementary Information file.
