# Supplementary material for: Control of Treg cell homeostasis and immune equilibrium by Lkb1 in dendritic cells
Source: Nat Commun. 2018 Dec 13;9:5298. doi: 10.1038/s41467-018-07545-8 (PMC6294005; doi:10.1038/s41467-018-07545-8)
Supplement: Supplementary file 1 — Supplementary Information [file 41467_2018_7545_MOESM1_ESM.pdf]

Chen, S et al.: Control of T_{reg} cell homeostasis and immune equilibrium by Lkb1 in dendritic cells

Supplementary materials: 17 Figures; 5 tables

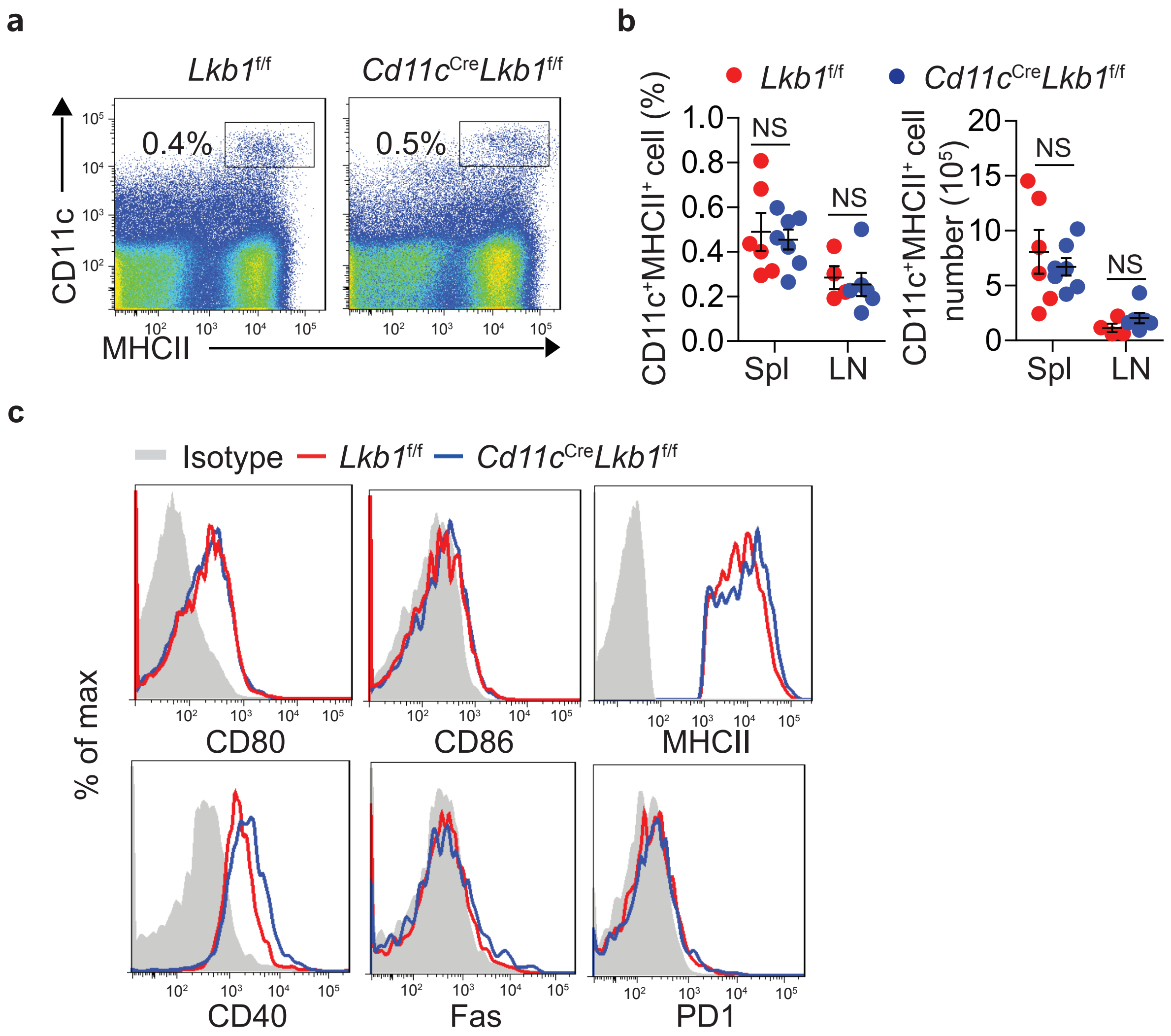
a
Lkb1f/f
Cd11cCreLkb1f/f
0.4%
0.5%
CD11c
MHCII
b
Lkb1f/f
Cd11cCreLkb1f/f
CD11c+MHCII+ cell (%)
CD11c+MHCII+ cell number (105)
NS
Spl
LN
c
Isotype
Lkb1f/f
Cd11cCreLkb1f/f
% of max
CD80
CD86
MHCII
CD40
Fas
PD1

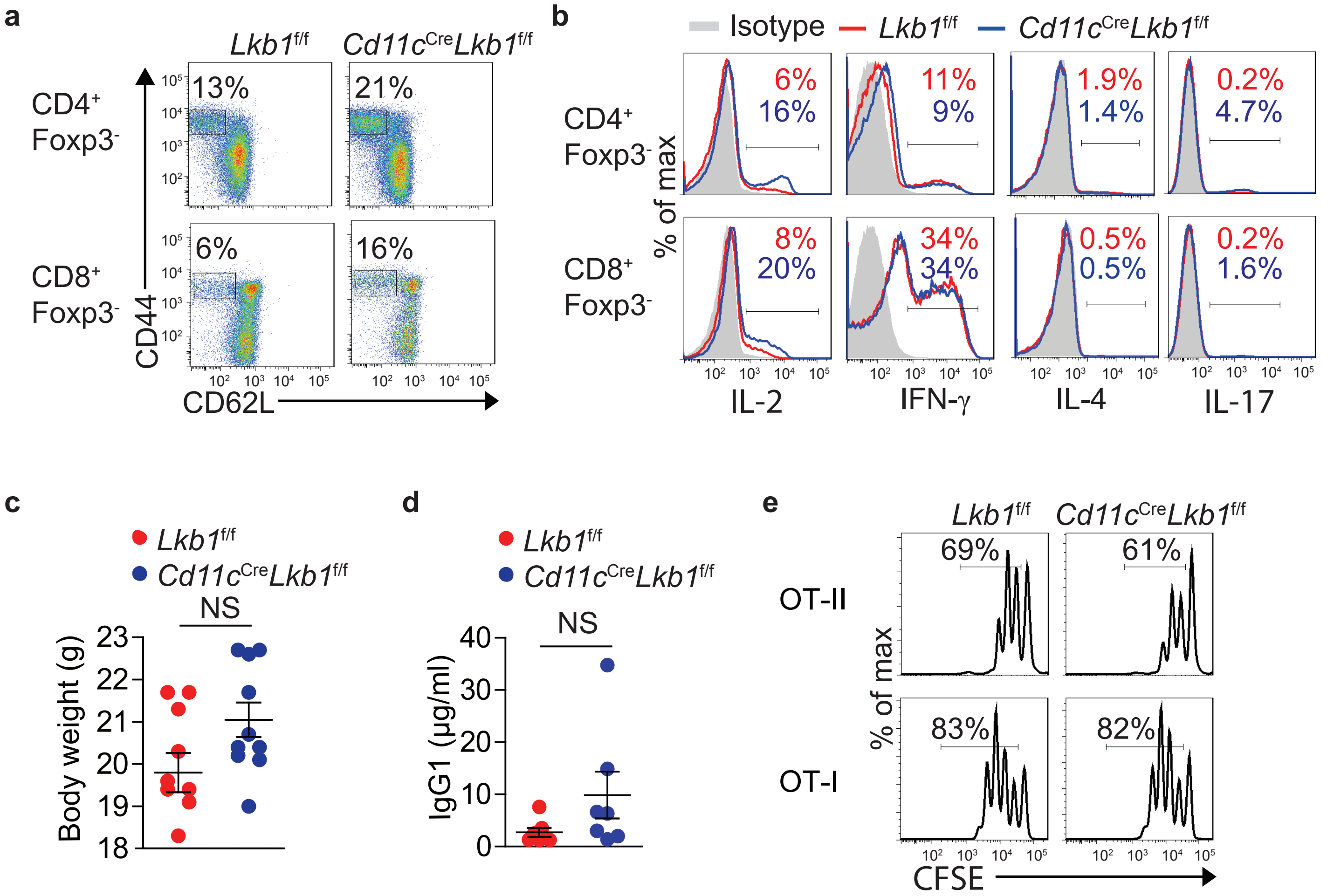
a
Lkb1f/f
Cd11cCreLkb1f/f
CD4+ Foxp3-
CD8+ Foxp3-
13%
21%
6%
16%
CD44
CD62L
b
Isotype
Lkb1f/f
Cd11cCreLkb1f/f
% of max
6%
16%
11%
9%
1.9%
1.4%
0.2%
4.7%
8%
20%
34%
34%
0.5%
0.5%
0.2%
1.6%
IL-2
IFN-γ
IL-4
IL-17
c
NS
Body weight (g)
d
NS
IgG1 (μg/ml)
e
OT-II
OT-I
69%
61%
83%
82%
CFSE

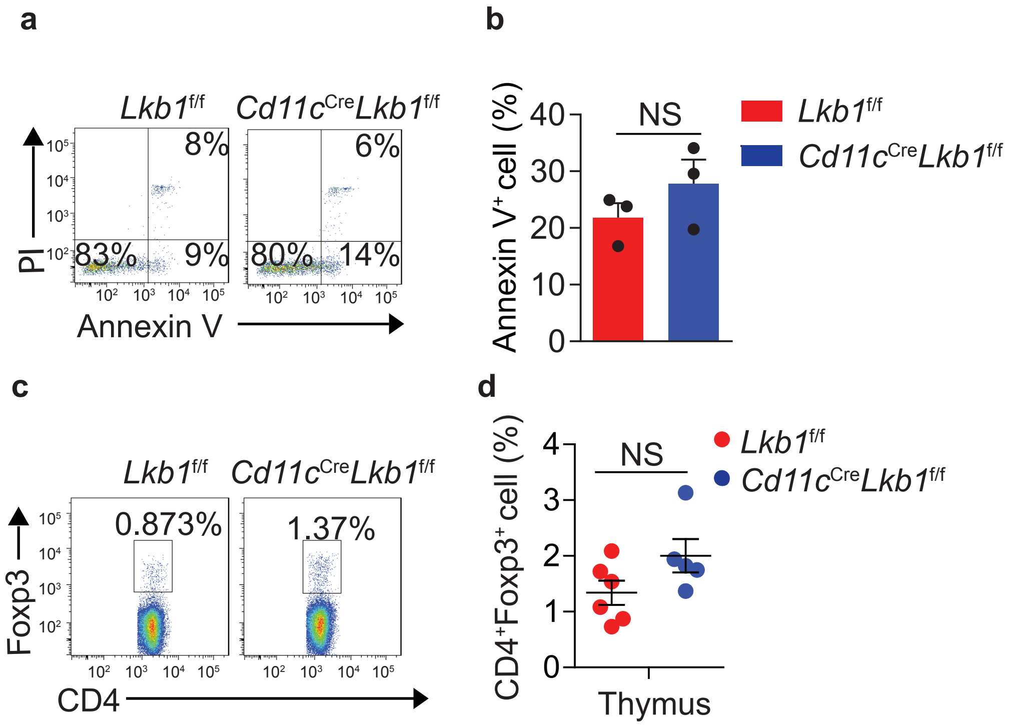

Supplementary Figure 3

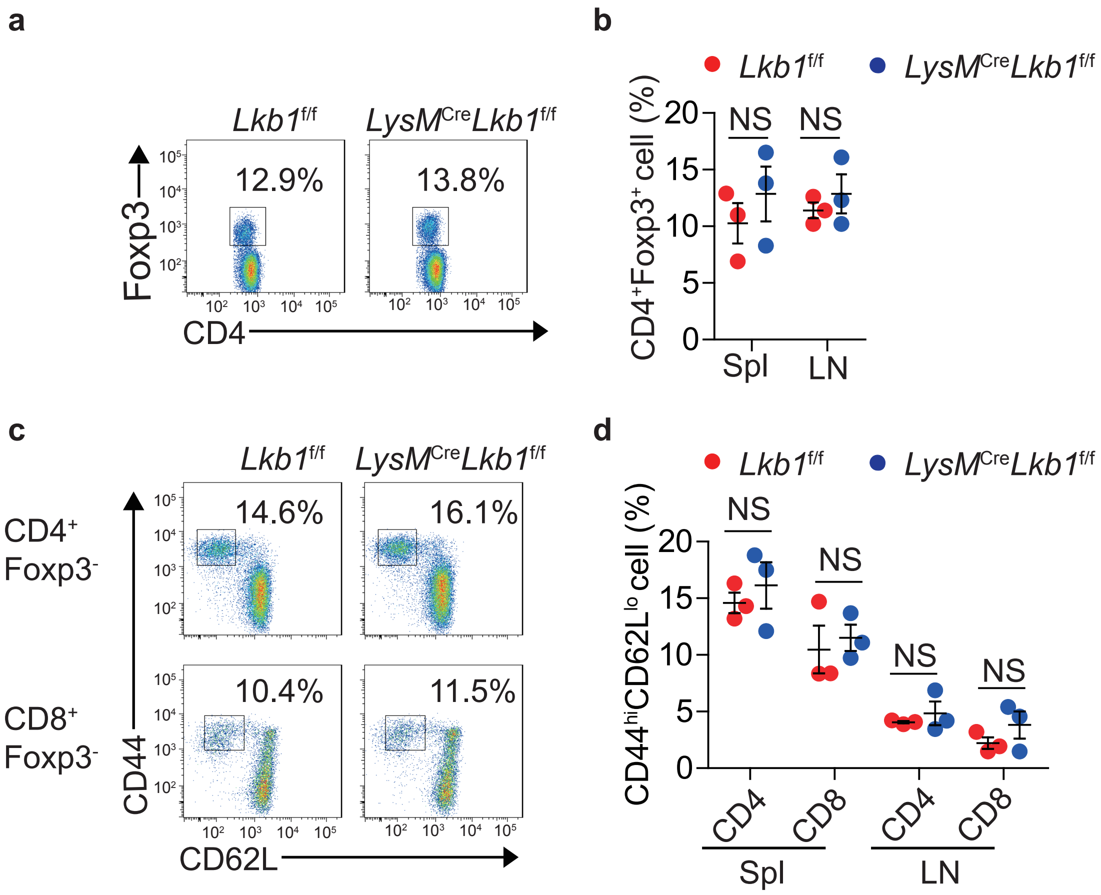

a
Lkb1f/f
LysMCreLkb1f/f
12.9%
13.8%
Foxp3
CD4
b
Lkb1f/f
LysMCreLkb1f/f
CD4+Foxp3+ cell (%)
NS
NS
Spl
LN
c
Lkb1f/f
LysMCreLkb1f/f
CD4+ Foxp3-
CD8+ Foxp3-
14.6%
16.1%
10.4%
11.5%
CD44
CD62L
d
Lkb1f/f
LysMCreLkb1f/f
CD44hiCD62Llo cell (%)
NS
NS
NS
NS
CD4
CD8
CD4
CD8
Spl
LN

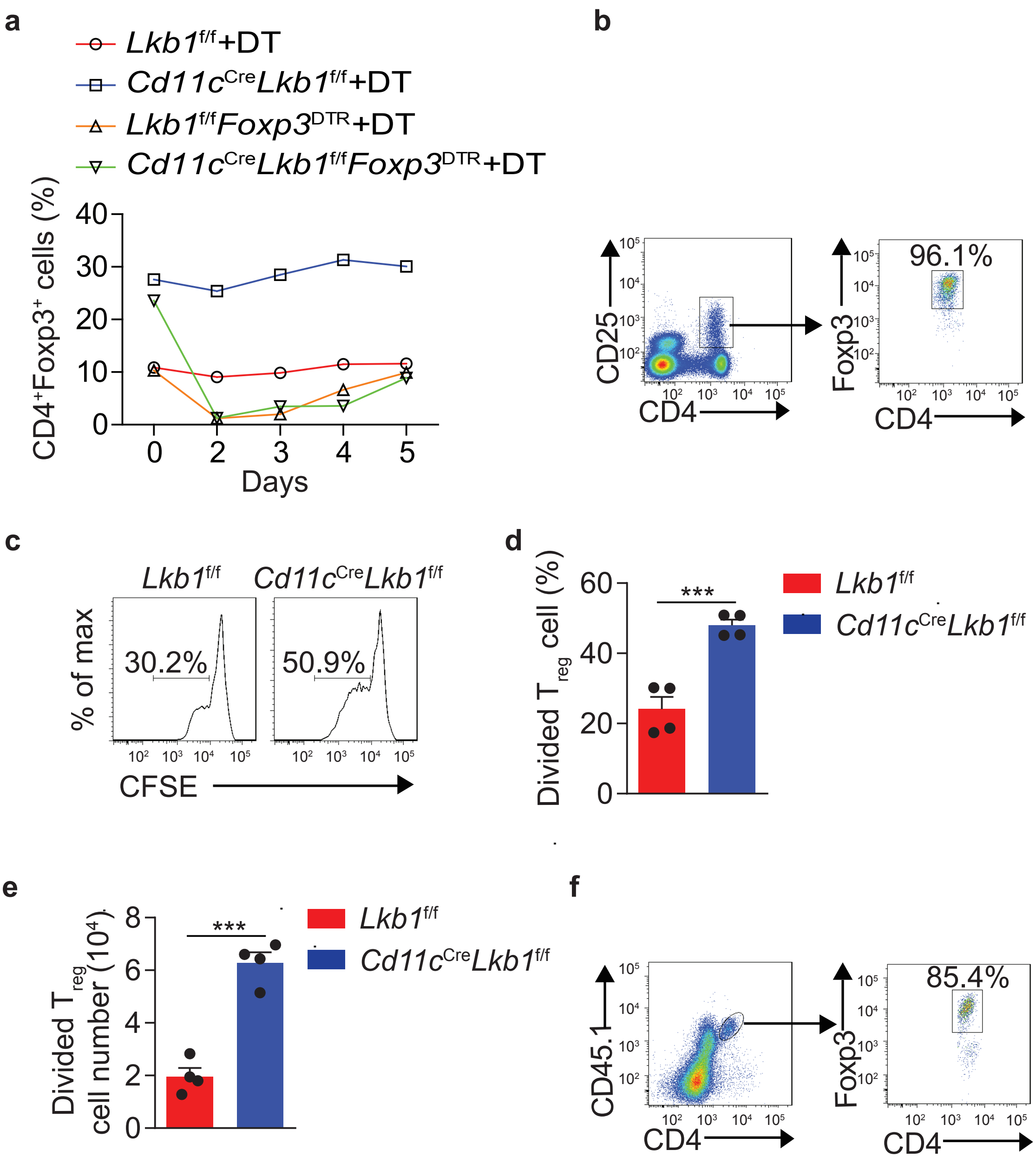

Supplementary Figure 5

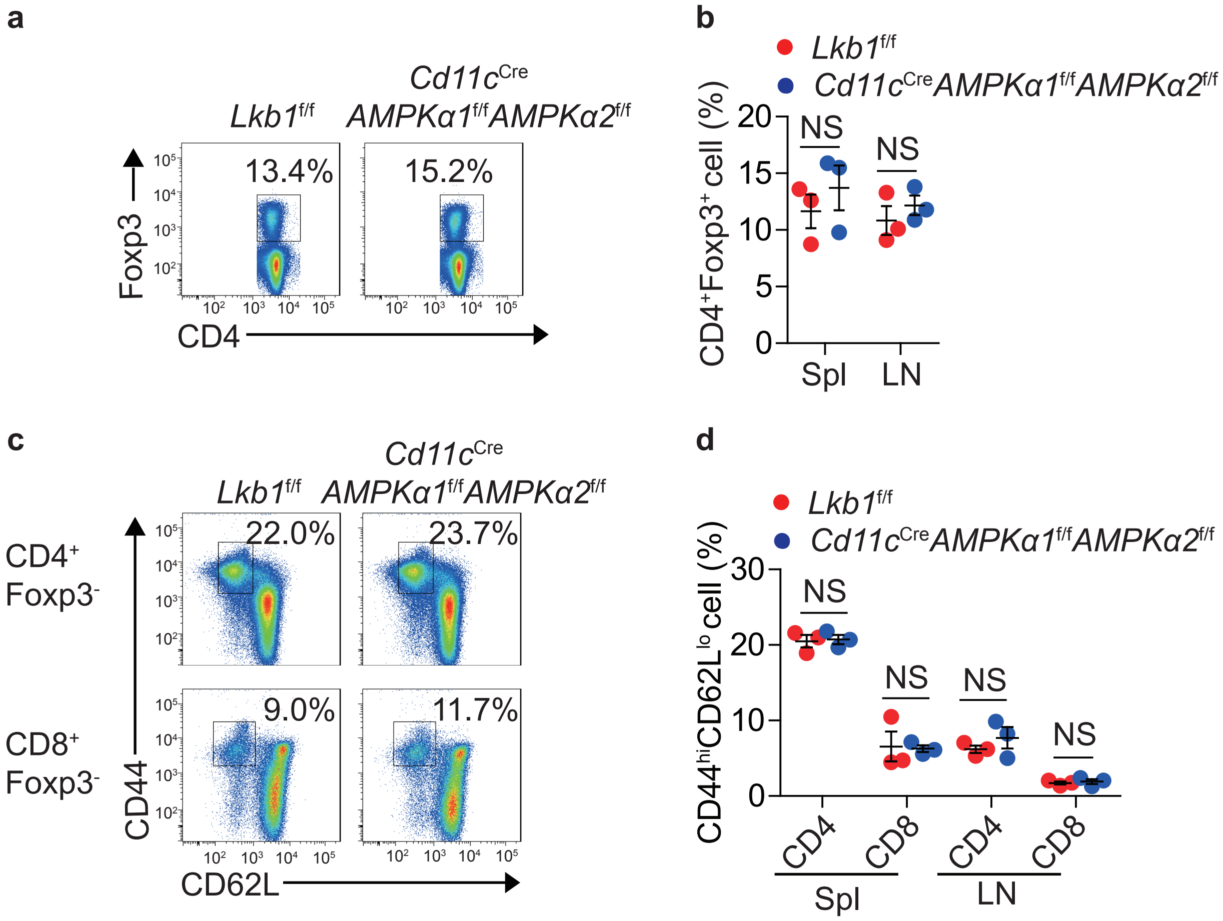

Supplementary Figure 6

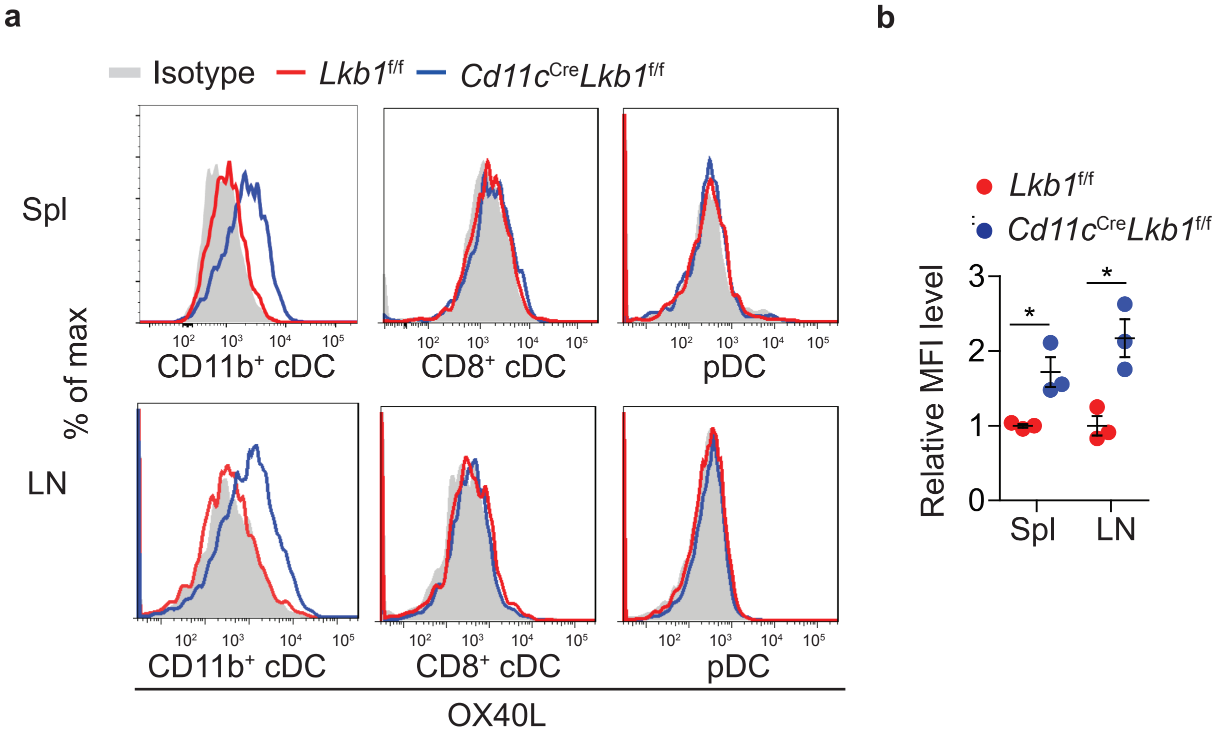
a
Isotype
Lkb1f/f
Cd11cCreLkb1f/f
Spl
LN
% of max
CD11b+ cDC
CD8+ cDC
pDC
OX40L
b
Relative MFI level
*
Spl
LN

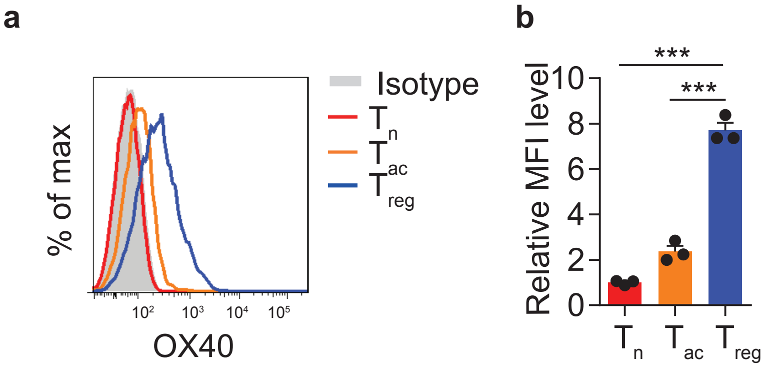

Supplementary Figure 8

a

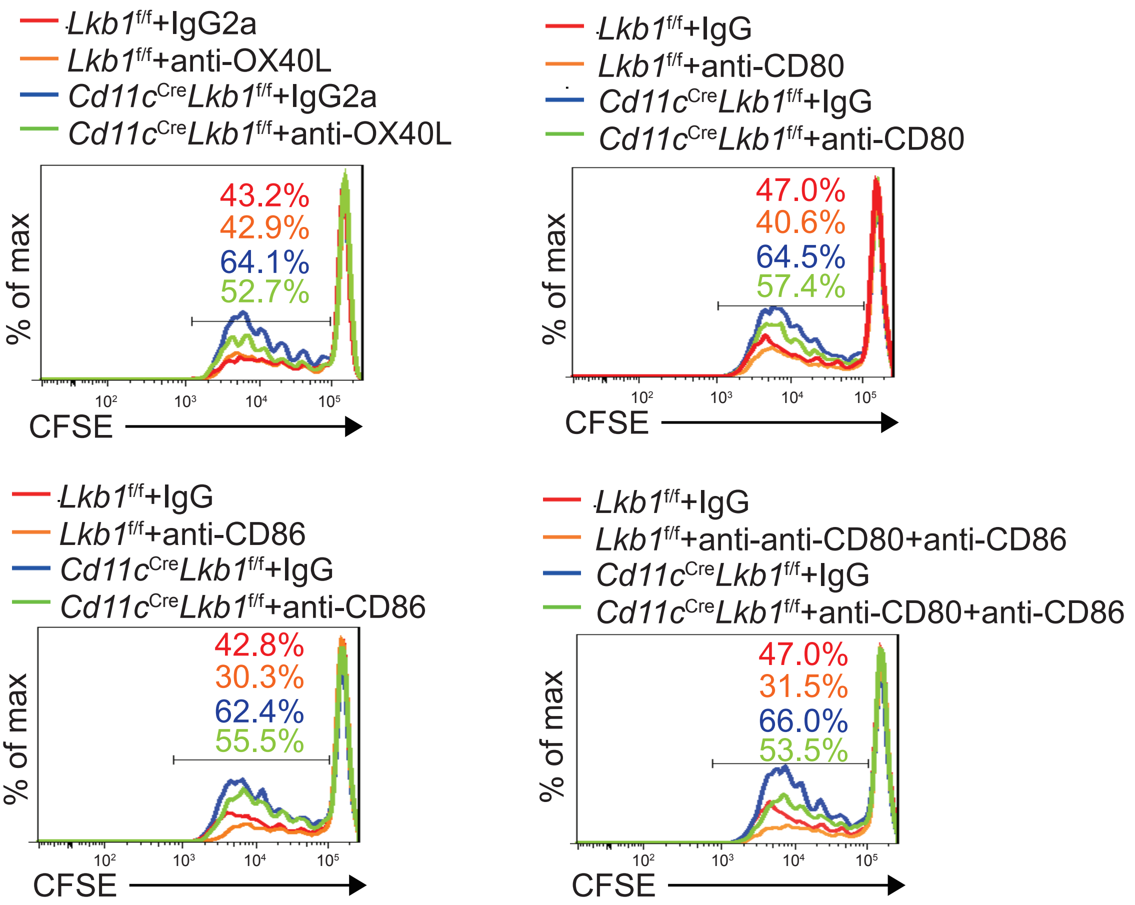

Lkb1$^{f/f}$+IgG2a
Lkb1$^{f/f}$+anti-OX40L
Cd11c^{Cre}*Lkb1*$^{f/f}$+IgG2a
Cd11c^{Cre}*Lkb1*$^{f/f}$+anti-OX40L
43.2%
42.9%
64.1%
52.7%
% of max
CFSE
Lkb1$^{f/f}$+IgG
Lkb1$^{f/f}$+anti-CD80
Cd11c^{Cre}*Lkb1*$^{f/f}$+IgG
Cd11c^{Cre}*Lkb1*$^{f/f}$+anti-CD80
47.0%
40.6%
64.5%
57.4%
% of max
CFSE
Lkb1$^{f/f}$+IgG
Lkb1$^{f/f}$+anti-CD86
Cd11c^{Cre}*Lkb1*$^{f/f}$+IgG
Cd11c^{Cre}*Lkb1*$^{f/f}$+anti-CD86
42.8%
30.3%
62.4%
55.5%
% of max
CFSE
Lkb1$^{f/f}$+IgG
Lkb1$^{f/f}$+anti-anti-CD80+anti-CD86
Cd11c^{Cre}*Lkb1*$^{f/f}$+IgG
Cd11c^{Cre}*Lkb1*$^{f/f}$+anti-CD80+anti-CD86
47.0%
31.5%
66.0%
53.5%
% of max
CFSE

b

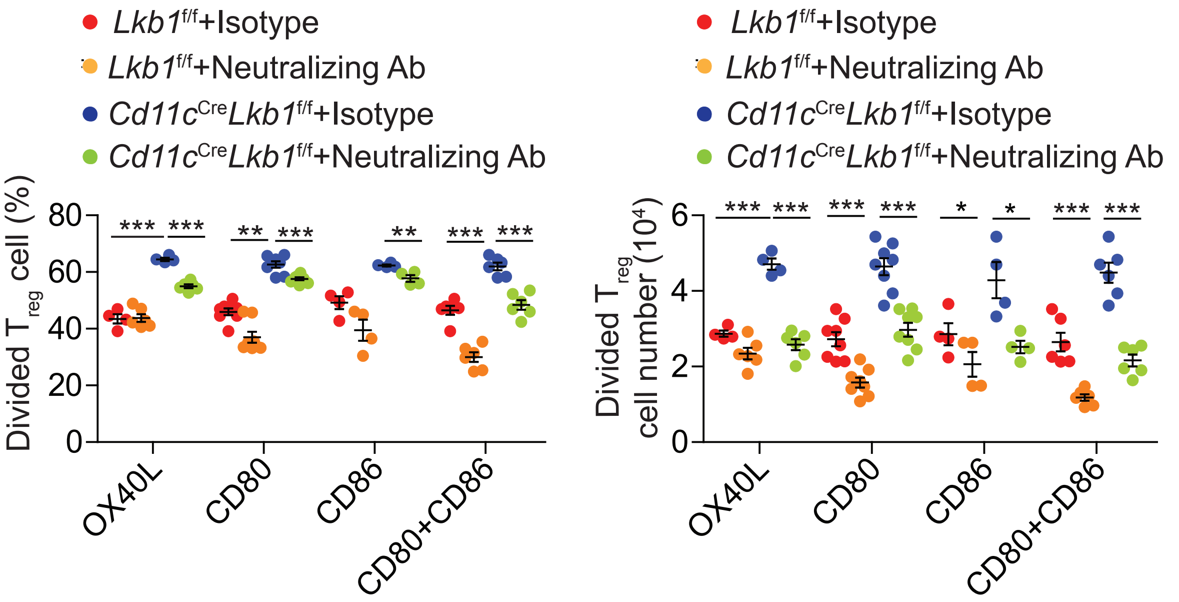

Lkb1$^{f/f}$+Isotype
Lkb1$^{f/f}$+Neutralizing Ab
Cd11c^{Cre}*Lkb1*$^{f/f}$+Isotype
Cd11c^{Cre}*Lkb1*$^{f/f}$+Neutralizing Ab
Divided T_{reg} cell (%)
Divided T_{reg} cell number (10^4)
OX40L
CD80
CD86
CD80+CD86

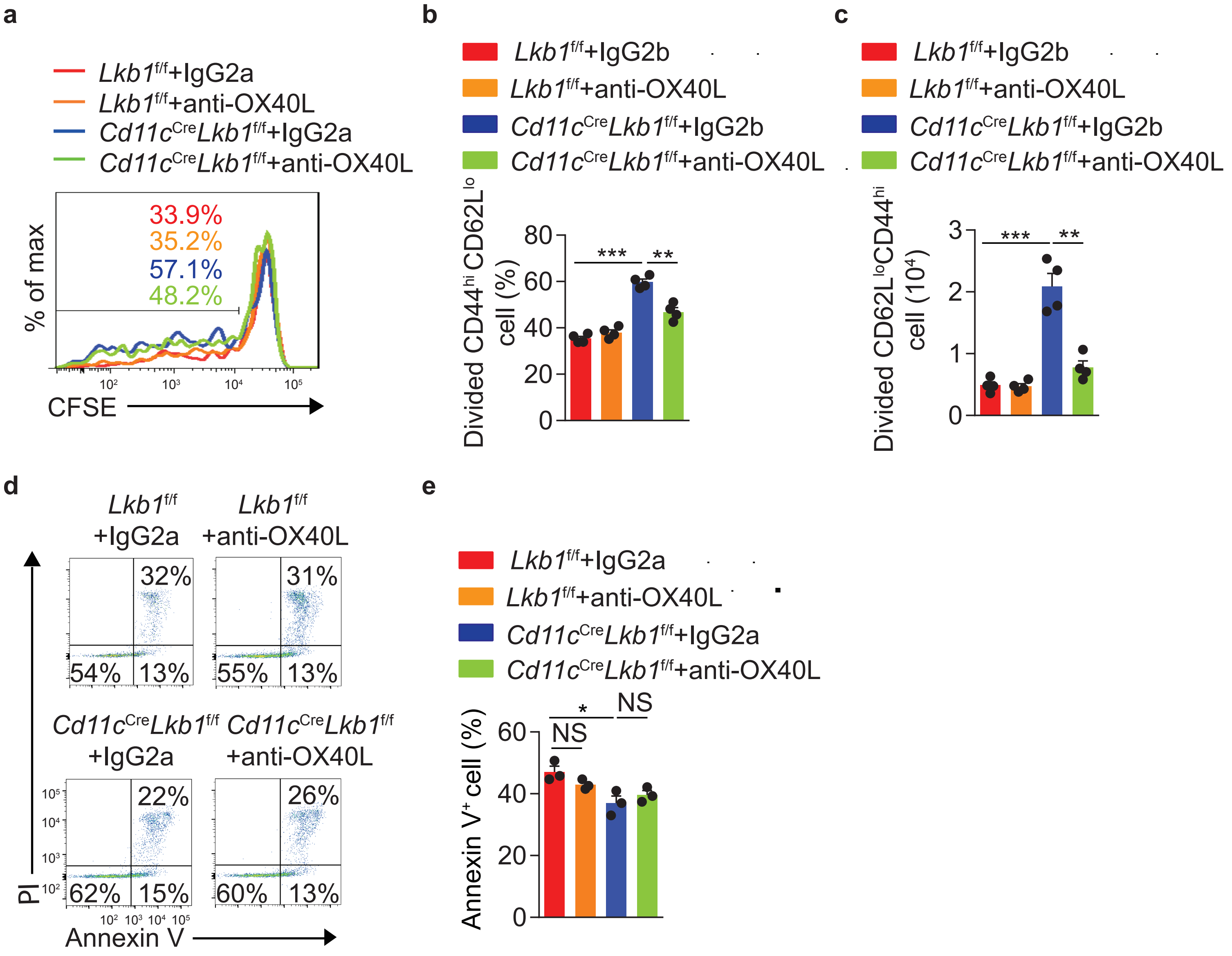

Supplementary Figure 10

a

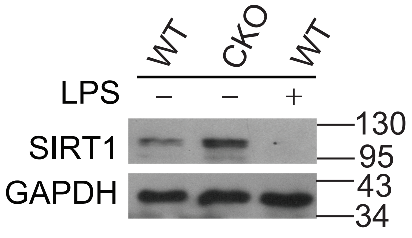

a

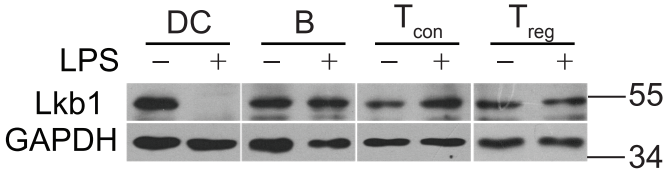

Supplementary Figure 12

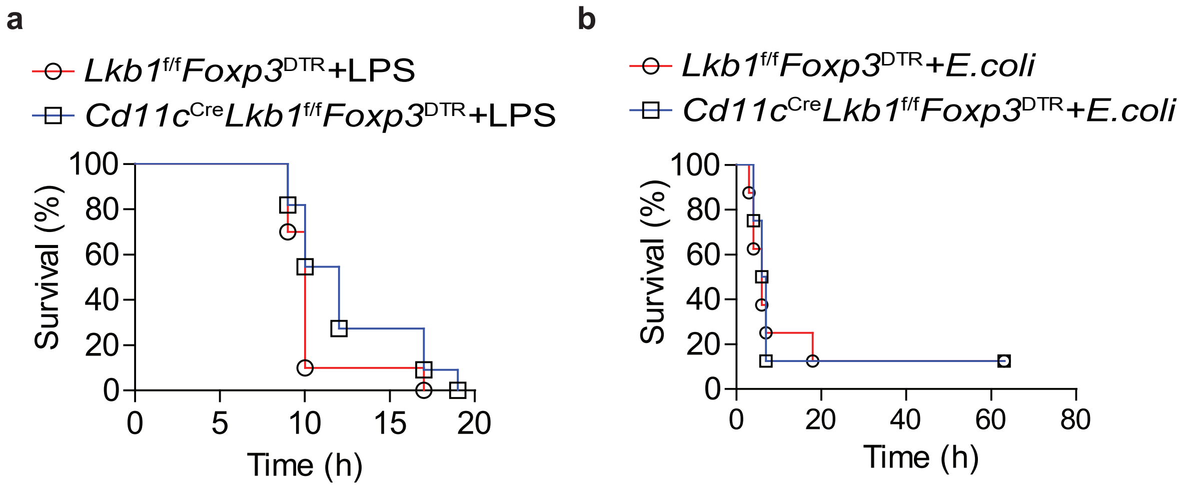

Supplementary Figure 13

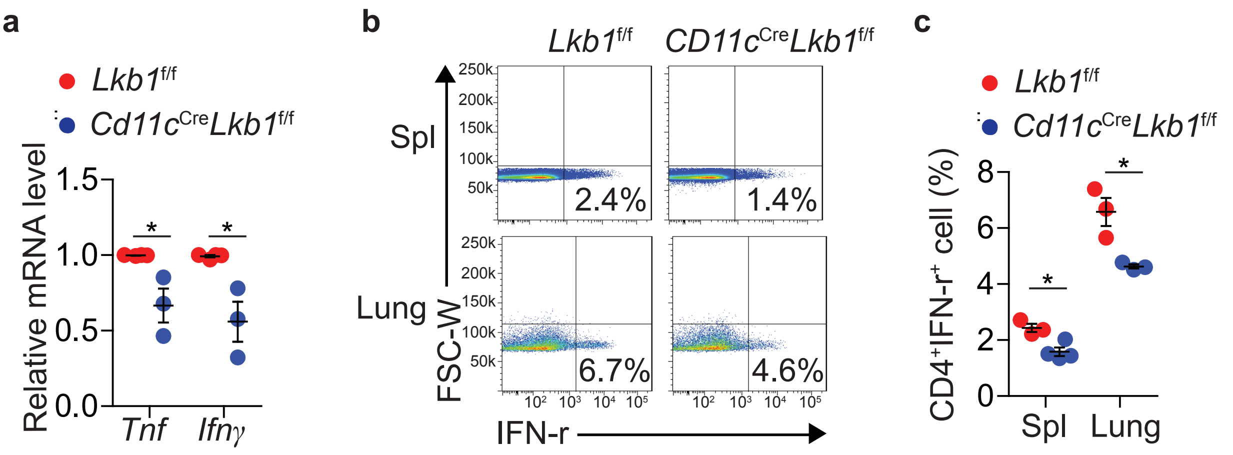

Supplementary Figure 14

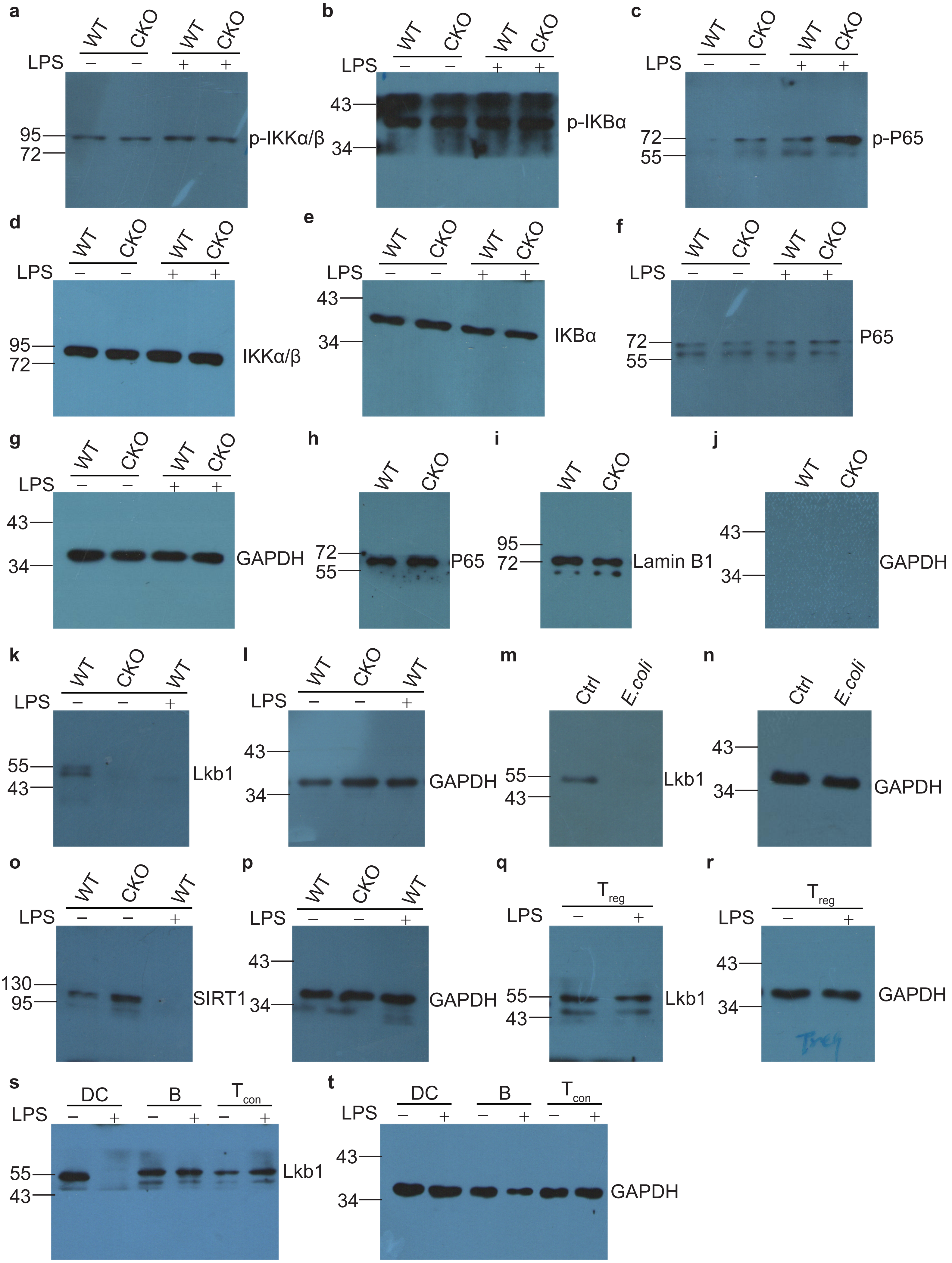

Supplementary Figure 15

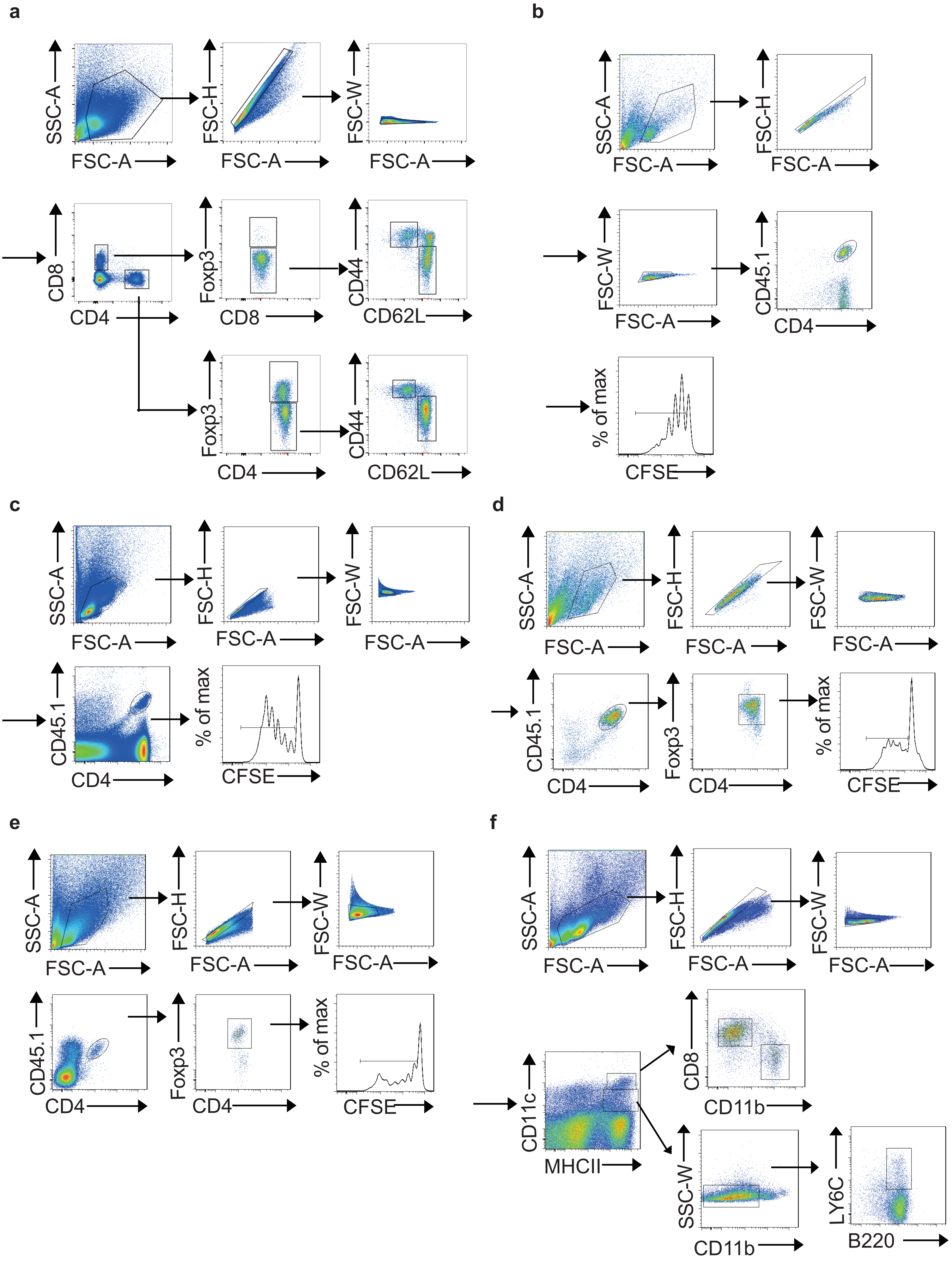

Supplementary Figure 16

a

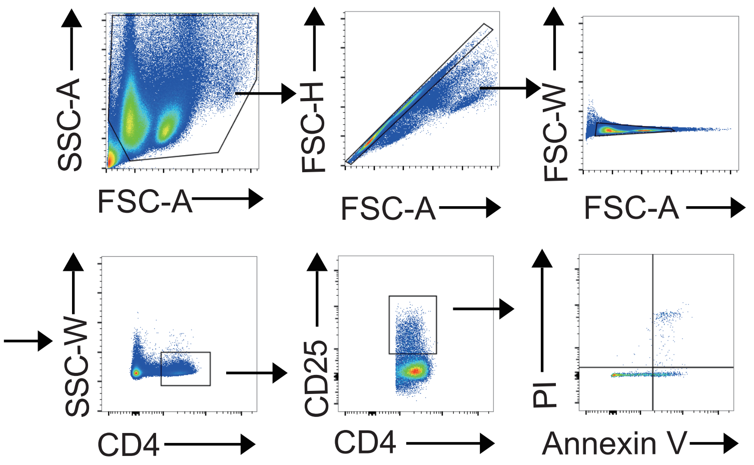

b

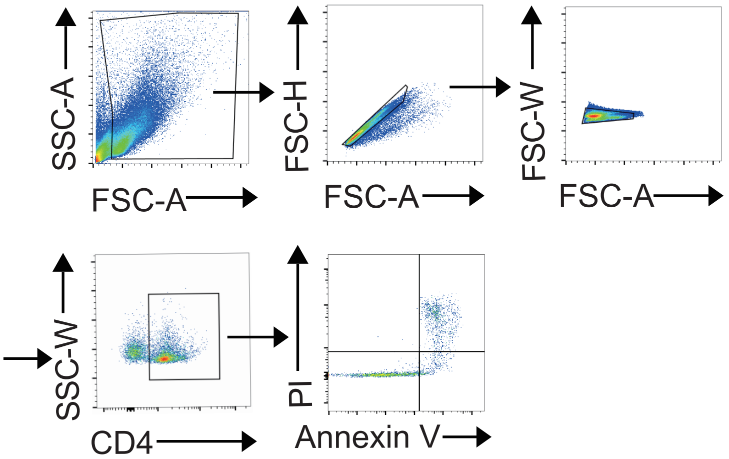

Supplementary Figure 17

Supplementary Table 1. Schematic description of conserved NF-κB binding site in *Ox40l* locus

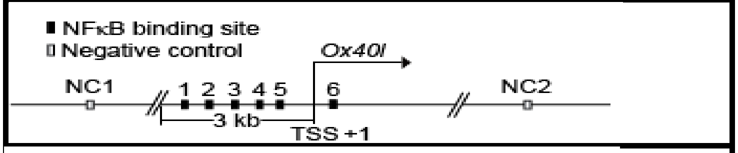

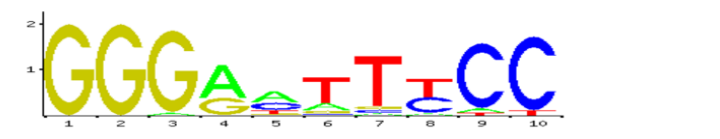

ID	Start	End	Strand	Site sequence
1	103	112	-1	GTGAATTACC
2	771	780	-1	AGGGTTTTCC
3	1821	1830	1	GAGACTTTCC
4	2441	2450	-1	GGGAAATTCA
5	2882	2891	-1	GTGGCATCCC
6	2945	2954	1	GGGAAATTCA
7	2967	2976	-1	GGGAACTTCT

Supplementary Table 2. Oligonucleotide primers

**Primer/Purpose**	**5'-Sequence-3'**
Ox40l /q-PCR of mouse *Ox40l* cDNA	F: GAAGAAGACGCTAAGGCTGGT<br>R: GAGCTGATGAATAGTTGCCCAT
Ox40 /q-PCR of mouse *Ox40* cDNA	F: TACCTACCCCAGTGGTCACAA<br>R: ACGGATGACATAGAGTATCCCTG
Lkb1 /q-PCR of mouse *Lkb1* cDNA	F: GCCAACGTCAAGAAGGAGAT<br>R: CGCAGTACTCCATCACCATATAC
Cxcl11 /q-PCR of mouse *Cxcl11* cDNA	F: GGCTTCCTTATGTTCAAACAGGG<br>R: GCCGTTACTCGGGTAAATTACA
Il1a /q-PCR of mouse *Il1a* cDNA	F: CGAAGACTACAGTTCTGCCATT<br>R: GACGTTTCAGAGGTTCTCAGAG
Il27 /q-PCR of mouse *Il27* cDNA	F: CTGTTGCTGCTACCCTTGCTT<br>R: CACTCCTGGCAATCGAGATTC
Cxcl9 /q-PCR of mouse *Cxcl9* cDNA	F: TCCTTTTGGGCATCATCTTCC<br>R: TTTGTAGTGGATCGTGCCTCG
Il15 /q-PCR of mouse *Il15* cDNA	F: ACATCCATCTCGTGCTACTTGT<br>R: GCCTCTGTTTTAGGGAGACCT
Cd80 /q-PCR of mouse *Cd80* cDNA	F: ACCCCCAACATAACTGAGTCT<br>R: TTCCAACCAAGAGAAGCGAGG
Cxcl10 /q-PCR of mouse *Cxcl10* cDNA	F: CCAAGTGCTGCCGTCATTTTC<br>R: GGCTCGCAGGGATGATTTCAA
Il1f9 /q-PCR of mouse *Il1f9* cDNA	F: TCCTGACTTTGGGGAGGTTTT<br>R: TCACGCTGACTGGGGTTACT
Ccl5 /q-PCR of mouse *Ccl5* cDNA	F: GCTGCTTTGCCTACCTCTCC<br>R: TCGAGTGACAAACACGACTGC
Gapgh /q-PCR of mouse *Gapgh* cDNA	F: AGGTCGGTGTGAACGGATTTG<br>R: TGTAGACCATGTAGTTGAGGTCA
Ox40l -NC1/ChIP-qPCR of mouse *Ox40l* gene	F: CTGGTCATGTGCTGATGAGT<br>R: TGAAGTTGGCTATGGTGAGAAT
Ox40l -1/ChIP-qPCR of mouse *Ox40l* gene	F: CCCACCAGAGTTGGTAATTCA<br>R: ATTTCAGTCCTGGGCAGATG
Ox40l -2/ChIP-qPCR of mouse *Ox40l* gene	F: CCACCATGAGGTTCTGAGTTT<br>R: CCCGTTTCAGCTTCCATAGT
Ox40l -3/ChIP-qPCR of mouse *Ox40l* gene	F: CTGTTGACGGCTCCATGATAAA<br>R: GCACAGGGATGCTGAACATAA
Ox40l -4/ChIP-qPCR of mouse *Ox40l* gene	F: GCTGAATTTCCCTGAAATGAGAAA<br>R: GAGCAACTGCTATGTGTCAGA
Ox40l -5/ChIP-qPCR of mouse *Ox40l* gene	F: TTCATAACTGGGATGCCACAA<br>R: GGGAACTTCTATGTGACTAATCTGAA
Ox40l -6/ChIP-qPCR of mouse *Ox40l* gene	F: CAAGTGGAAGAAGACGCTAAGG<br>R: CTTACCGGAGAGGAAGAGAGTT
Ox40l -NC2/ChIP-qPCR of mouse *Ox40l* gene	F: ACTTCCAGTGCTTTCCTTACA<br>R: GCTGACCAAGATAGCCAATTTC

Supplementary Table 3. Flow cytometry gating strategies.

Tissue/ cells	Target Population	Gating Strategy	Figure number
Spleen, lymph nodes, thymus, lung and brain	$CD4^+Foxp3^+$ T_{reg} cells	Immune cells/Singlets/$CD4^+$/$Foxp3^+$	Supplementary Figure 16a-> Figure 1a,1c,1e,1g,6c,6e,7b,7d and Supplementary Figure 3c,4a,6a,8a
	$CD4^+Foxp3^-$ T cells	Immune cells/Singlets/$CD4^+$/$Foxp3^-$	Supplementary Figure 16a-> Figure 2b and Supplementary Figure 2a,2b,4c,6c,8a,14b
	$CD8^+Foxp3^-$ T cells	Immune cells/Singlets/$CD8^+$/$Foxp3^-$	Supplementary Figure 16a-> Supplementary Figure 2a,2b,4c,6c
Co-cultured cells	$CD4^+CD45.1^+$ T cells	Immune cells/Singlets/$CD4^+CD45.1^+$	Supplementary Figure 16b-> Figure 1h and Supplementary Figure 2e,10a
Spleen	$CD4^+CD45.1^+$ T cells	Immune cells/Singlets/$CD4^+CD45.1^+$	Supplementary Figure 16c-> Figure 2d,2g
Co-cultured cells	$CD4^+CD45.1^+Foxp3^+$ T_{reg} cells	Immune cells/Singlets/$CD4^+CD45.1^+$/$Foxp3^+$	Supplementary Figure 16d-> Figure 3a,3d,3g,4e,5e,6g and Supplementary Figure 9a
Spleen	$CD4^+CD45.1^+Foxp3^+$ T_{reg} cells	Immune cells/Singlets/$CD4^+CD45.1^+$/$Foxp3^+$	Supplementary Figure 16e-> Figure 3h,4h,5h
Spleen and lymph nodes	$MHCII^+CD11c^+$ DCs	Immune cells/Singlets/$MHCII^+CD11c^+$	Supplementary Figure 16f-> Figure 4d,5d,8e and Supplementary Figure 1a,1c,
	$MHCII^+CD11c^{hi}CD11b^+$ cDCs	Immune cells/Singlets/$MHCII^+CD11c^{hi}$/$CD8^-CD11b^+$	Supplementary Figure 16f-> Supplementary Figure 7a
	$MHCII^+CD11c^{hi}CD8^+$ cDCs	Immune cells/Singlets/$MHCII^+CD11c^{hi}$/ $CD8^+CD11b^-$	Supplementary Figure 16f-> Supplementary Figure 7a
	$MHCII^+CD11c^{low}CD11b^-B220^+LY6C^+$ pDCs	Immune cells/Singlets/$MHCII^+CD11c^{low}$/$CD11b^-$/ $B220^+LY6C^+$	Supplementary Figure 16f-> Supplementary Figure 7a
Spleen	$CD4^+CD25^+$ T_{reg} cells	Immune cells/Singlets/ $CD4^+$/$CD25^+$	Supplementary Figure 17a-> Supplementary Figure 3a
Co-cultured cells	$CD4^+$ T cells	Immune cells/Singlets/$CD4^+$	Supplementary Figure 17b-> Supplementary Figure 10d
