# Supplementary material for: Control of Treg cell homeostasis and immune equilibrium by Lkb1 in dendritic cells
Source: Nat Commun. 2018 Dec 13;9:5298. doi: 10.1038/s41467-018-07545-8 (PMC6294005; doi:10.1038/s41467-018-07545-8)
Supplement: Supplementary file 2 — Description of Additional Supplementary Files [file 41467_2018_7545_MOESM2_ESM.pdf]

Description of Additional Supplementary Files

**Supplementary Data 1.** Gene expression alterations in Lkb1-deficient DCs (≥1.5 fold change)

**Supplementary Data 2.** Gene expression alterations in LPS-treated DCs (≥1.5 fold change)
